# *Bcor* deficiency perturbs erythro*-*megakaryopoiesis and cooperates with *Dnmt3a* loss in acute erythroid leukemia onset in mice

**DOI:** 10.1038/s41375-020-01075-3

**Published:** 2020-11-06

**Authors:** Paolo Sportoletti, Daniele Sorcini, Anna G. Guzman, Jaime M. Reyes, Arianna Stella, Andrea Marra, Sara Sartori, Lorenzo Brunetti, Roberta Rossi, Beatrice Del Papa, Francesco Maria Adamo, Giulia Pianigiani, Camilla Betti, Annarita Scialdone, Valerio Guarente, Giulio Spinozzi, Valentina Tini, Maria Paola Martelli, Margaret A. Goodell, Brunangelo Falini

**Affiliations:** 1 Centro di Ricerca Emato-Oncologica (CREO), University of Perugia, Perugia, 06132 Italy; 2 Stem Cell and Regenerative Medicine, Baylor College of Medicine, Houston, TX 77030 USA; 3 Center for Cell and Gene Therapy, Texas Children’s Hospital and Houston Methodist Hospital, Baylor College of Medicine, Houston, TX 77030 USA

**Keywords:** Acute myeloid leukaemia, Preclinical research

## Abstract

Recurrent loss-of-function mutations of *BCL6 co-repressor* (*BCOR)* gene are found in about 4% of AML patients with normal karyotype and are associated with *DNMT3a* mutations and poor prognosis. Therefore, new anti-leukemia treatments and mouse models are needed for this combinatorial AML genotype. For this purpose, we first generated a *Bcor*^*−/−*^ knockout mouse model characterized by impaired erythroid development (macrocytosis and anemia) and enhanced thrombopoiesis, which are both features of myelodysplasia/myeloproliferative neoplasms. We then created and characterized double *Bcor*^*−/−*^*/Dnmt3a*^*−/−*^ knockout mice. Interestingly, these animals developed a fully penetrant acute erythroid leukemia (AEL) characterized by leukocytosis secondary to the expansion of blasts expressing c-Kit+ and the erythroid marker Ter119, macrocytic anemia and progressive reduction of the thrombocytosis associated with loss of *Bcor* alone. Transcriptomic analysis of double knockout bone marrow progenitors revealed that aberrant erythroid skewing was induced by epigenetic changes affecting specific transcriptional factors (GATA1-2) and cell-cycle regulators (Mdm2, Tp53). These findings prompted us to investigate the efficacy of demethylating agents in AEL, with significant impact on progressive leukemic burden and mice overall survival. Information gained from our model expands the knowledge on the biology of AEL and may help designing new rational treatments for patients suffering from this high-risk leukemia.

## Introduction

The *BCL6 co-repressor* (*BCOR)* gene is located on chromosome Xp11.4 and encodes a transcription regulatory factor that was initially identified as an interactor partner of the germinal center-associated BCL6 protein [1, 2]. The BCOR protein is located in the nucleus [3] where exerts its function as a member of the non-canonical multimeric polycomb group repressive complex 1 (PRC1) which is recruited to the target sites independently of H3K27me3 [4]. This complex is involved in the control of various biological processes, including pluripotency, reprogramming, and hematopoiesis [4, 5]. Relatively high frequency of *BCOR* mutations has been reported in aplastic anemia suggesting that these genetic events may confer a selective advantage in the context of aplastic anemia autoimmune environment, although they do not appear to be associated with an increased risk of secondary AML/MDS [6]. Moreover, various aberrations of the *BCOR* gene, such as internal tandem duplications of the PCGF Ub-like fold discriminator domain, gene fusions, and loss-of-function mutations play a role in promoting hematological and extra-hematological malignancies [7, 8].

In 2011, we discovered recurrent loss-of-function mutations of *BCOR* in AML (about 4% of cases with normal karyotype) and found that they were usually mutually exclusive of *FLT3-ITD* and *NPM1* mutations, co-occurred with DNA methyl transferase (*DNMT3A*) mutations and were associated with a poor outcome [9]. Our findings have been subsequently confirmed by other investigators both in AML and MDS [10–14]. Interestingly, one study on Japanese patients reported a preferential co-occurrence of *BCOR* mutations with *K-RAS*, *N-RAS,* and *RUNX1* mutations [11]. *BCOR 1 ligand (BCORL1*) gene has been also found to be mutated in 3.7–6% of AML patients [11, 15].

*Bcor* somatic heterozygous mutations in AML are similar to the germline mutations that in females cause rare genetic syndrome characterized by cranio-facial, ocular and cardiac abnormalities [16]. The disruptive nature of these mutations that usually results in a premature stop codon and non-sense mediated decay or protein truncation are consistent with a tumor-suppressive role of the *Bcor* gene in myeloid malignancies. Accordingly, mice lacking *Bcor* exons 9 and 10, which encode for a carboxyl-terminal truncated *Bcor* unable to interact with the PCR1 core effector components, are characterized by expansion of myeloid progenitors [17], enhanced cell proliferation, and myeloid differentiation associated with upregulation of *HoxA* cluster [18].

However, *Bcor* deficiency is not itself sufficient to promote leukemia [17, 18], strongly suggesting that other mutations are required to induce myeloid malignancies. The type of myeloid neoplasm developing in *Bcor*-deficient mouse may vary depending on the co-occurring genetic lesions. For example, compound mice carrying concurrent full deletion of *Tet2* develop lethal MDS [19] whilst *Bcor* loss cooperates with Kras^G12D^ to drive AML [17].

We previously found that about 40% of AML patients with *BCOR* mutations also carry mutations of *DNMT3a* [9] catalyzing the addition of methyl groups to CpG dinucleotides. Because *BCOR* and *DNMT3A* are both epigenetic modifiers [4, 20, 21], mutations of these genes could promote AML through a synergistic mechanism [9]. Murine hematopoietic cells lacking *Dnmt3a* have thousands of focal, “canonically” located, hypomethylated regions that are amenable to be “repaired” with partial correction of dysregulated gene expression and myeloid skewing [22]. *Dnmt3a* loss with its hypomethylated phenotype is known to promote expansion and immortalization of hematopoietic stem cells, block in hematopoietic differentiation [23] and development of myeloid and lymphoid malignancies after a long period of latency [23–25]. Mx1-Cre-mediated *Dnmt3a* ablation led to the development of a lethal, fully penetrant myelodysplasia/myeloproliferative (MDS/MPN) neoplasm characterized by peripheral cytopenias and marked extramedullary hematopoiesis with liver involvement [26]. Thus, *Dnmt3a* deficiency establish an epigenetic state somehow predisposing to the emergence of cooperating mutations leading to overt leukemia in mice [25]. Indeed, co-expression of mutated *Dnmt3a* with other disease alleles (including *Tet2*, *Flt3*, *Npm1*) promote overt leukemic transformation in mice [2, 27]. However, information on the cooperation between *Dnmt3a* and *Bcor* is still missing.

Therefore, we generated a conditional mouse model of *Bcor* inactivation to explore its function in normal and leukemic hematopoiesis either alone or in combination with *Dnmt3a* loss. *Bcor*-deficient mice demonstrated impaired erythroid development and enhanced thrombopoiesis. Notably, the *Bcor*^*−/−*^*Dnmt3a*^*−/−*^ double knockout mice developed a fully penetrant acute erythroid leukemia (AEL) sensitive to demethylating agents. Information gained from this model expand our knowledge on the biology of AEL and may help to design new rational treatments for patients suffering from this high-risk leukemia.

## Methods

### Mouse strains

Mice were bred and housed by the “Service center of Preclinical Research” of Perugia’s animal house facility, and mouse manipulations were performed according to the protocol reviewed and approved by the Italian Health Ministry (generation on Supplementary Methods).

### Peripheral blood counts

Mice were anesthetized with isoflurane followed by retro-orbital bleeding. Peripheral blood (PB) was taken into glass capillary tubes. Complete blood count was performed using an XE-2100 hematology automated analyzer (Dasit).

### Flow Cytometry and Cell Sorting

Bone marrow, spleen, and PB cells were stained with antibodies from eBioscience (see list on Supplementary Methods). Cell acquisition and analysis were performed on BD FACS CANTO and BD FORTESSA. Sorting experiments (LSK and MEP subpopulations) were performed using the FACS AriaIII cell sorter. Gates were drawn to exclude nonviable cells and debris. Part of the flow cytometry data was analyzed with FlowJo software (Tree Star, Ashland, OR).

### Histology and cytospins

BM, spleen, liver, kidney, lung, and spinal cord were harvested from moribund mice. Spinal cord was decalcified, then spleen, liver, kidney, lung, and spinal cord were paraffin embedded, and sections were stained with hematoxylin and eosin and cytospins were stained with Giemsa. Cytospins of BM and spleen were performed both before and after red blood cell (RBC) lysis.

### Gene expression profiling and accession numbers

Total RNA was extracted from LSK and MEP BM cells using an RNA extraction kit (RNeasy plusMicro kit, Qiagen) and samples analyzed for Gene Expression Profiling. Starting from the results of the differential analysis, the comparisons of all three mutated genotypes with the wild type were taken into consideration and the genes were filtered considering only those with a *p* value < 0.05 and an absolute fold change value >1.5.

The three resulting lists were compared to evaluate both common and exclusive genes for each genotype. To show these similarities and differences we have resorted to Venn diagrams (GSE158018).

### In vivo treatments

Mice were treated through intra-peritoneal injections with a maximum tolerated dose of the demethylating agent 5-aza-2’-deoxycytidine DEC (2.5 mg/kg, every 3 days 5 doses) and Cytarabine ARA-C (50 mg/kg for 6 days).

### Immunoblotting analysis

Gata1 protein was detected by western blot analysis on lysates from 1 to 2 × 10^6^ cells with an anti-rat primary antibody against GATA-1 (N6) clone sc-265 (Santa Cruz biotechnology). After washing, blots were incubated with HPR-conjugated secondary antibody anti-IgG (Sigma). Mouse a-tubulin antibody was used as control and obtained from Sigma.

## Results

### Loss of *Bcor* induces red blood cells changes and expansion of the megakaryocyte compartment

We generated a conditional knockout mouse model in which *Bcor* deletion mimics truncating *Bcor* mutations observed in AML. Our *Bcor* conditional knockout mouse model was developed deleting exons from 8 to 10, resulting in frameshift and premature stop codon in exon 11. Originated mRNA preserves the polyA tail but is extremely unstable due to the presence of five splicing junctions between the STOP codon and the polyA tail (Supplementary Fig. 1A, B). To test the impact of *Bcor* deletion in adult hematopoiesis, mutant mice were crossed with *Mx1-Cre* mice carrying an interferon-inducible Cre recombinase transgene under an hematopoietic stem cell promoter. PCR genotyping of the offspring allowed the identification of both the wild type and mutant alleles from tails DNA (Supplementary Fig. 1Ci) and the recombination of loxP-containing target alleles in *Bcor-flox/flox*-*Cre* + BM after pIpC induction of the Cre (Supplementary Fig. 1Cii). To confirm that the induced *Mx1-Cre* + *Bcor* mutant mice did not express *Bcor* mRNA, RNA from BM Lin-Sca+c-Kit + (LSK) was reverse transcribed and amplified by PCR. *Bcor* mRNA was not detectable by this method in *Bcor-flox/flox*-*Cre* + *(Bcor*^*−/−*^) homozygous and *Bcor-flox/Y*-*Cre* + *(bcor*^*−/−*^) hemyzigous mice (Supplementary Fig. 2Ai). Western blot analysis of BM lin- cells from *Bcor-flox/flox*-*Cre* + *(Bcor*^*−/−*^) homozygous and hemyzigous demonstrated loss of Bcor protein (Supplementary Fig. 2Aii).

**Fig. 1:**
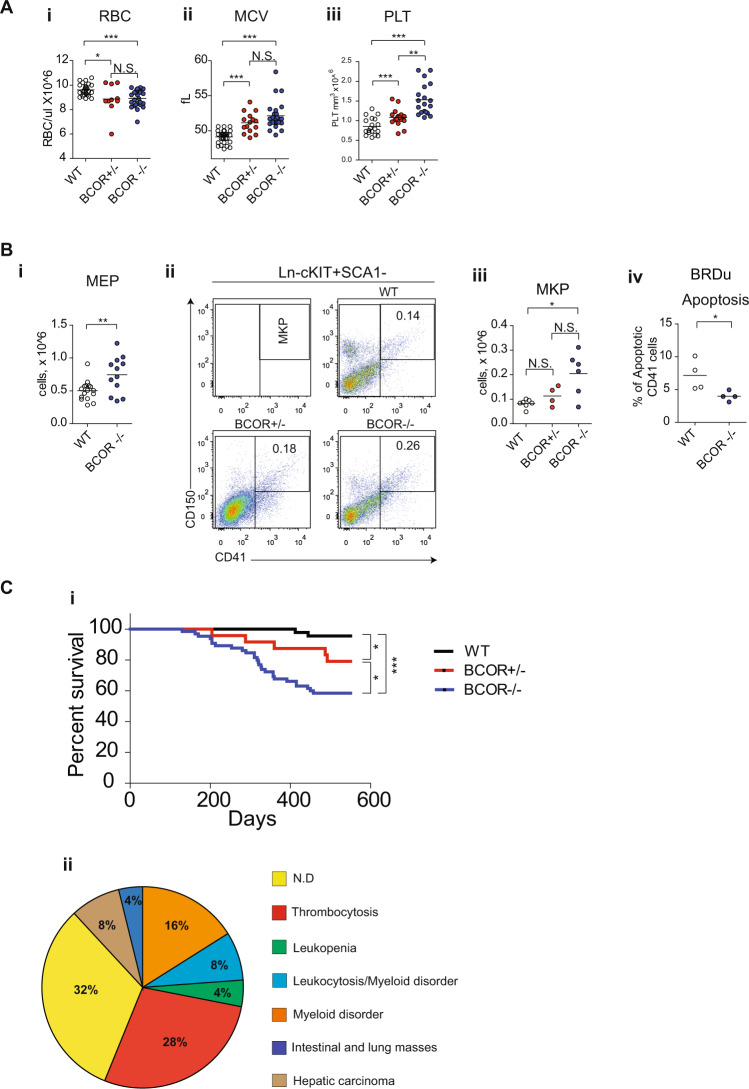
Loss of *Bcor* induces hematopoietic changes in mice. **A** RBCs count (i) mean corpuscolar volume (MCV) (ii) and platelets count (iii) in PB of *Bcor*^*−/−*^, *Bcor*^*+/−*^ and WT from 12 months old mice. **B** (i) Megakaryocyte-erythroid progenitors (MEP Lin − /Sca1-/Kit+CD34-FCgRII/IIIlo/-) in BM of *Bcor*^*−/−*^ and WT 6 months old mice. (ii) Representative flow-cytometric analysis of immature megakaryocytic compartments, megakaryocyte progenitor (MkP) and total number of MkP (iii) in BM of *Bcor*^*−/−*^, *Bcor*^*+/−*^ and WT mice. (iv) total number of CD41 + cells in BM of *Bcor*^*−/−*^, *Bcor*^*+/−*^ and WT 6 months old mice. (iv) Apoptosis in CD41 cells in WT and *Bcor*^*−/−*^ 3 months old mice. 1 × 10^6^ cells from BM were plated for 6 h in RPMI + BRDU, than counted and stained. **C** (i) Kaplan–Mayer plot of mouse survival according to the indicated genotypes (*n* = 44 WT,19 *Bcor*^*+/−*^, 37 *Bcor*^*−/−*^*)*. (ii) Pie charts showing the different causes of mortality: 56% of deaths occurred in the *Bcor* mutant cohort in the presence of well-defined hematological abnormalities. In the remaining mice, pathological examinations were consistent with the occurrence of hepatic carcinoma (8%), intestinal/lung tumors (4%), possibly due to Cre-recombinase leakiness. Notably, 32% of deaths were of unknown origin. *p < 0.05, **p < 0.01; ***p < 0.001 unpaired *t*-test with Welch’s correction.

To determine the effect of *Bcor* loss in mice hematopoietic system, we performed serial complete blood counts that showed leukopenia (mainly due to B-cell lymphopenia (Supplementary Fig. 3Ai–v), red blood cells’ (RBC) reduction (Fig. 1Ai) with increased mean corpuscle volume (MCV) (Fig. 1Aii), and platelet counts’ progressive increase (Fig. 1Aiii; Supplementary Fig. 3B, C). Resulting thrombocytosis derived from the accumulation of both megakaryocytic-erythroid (MEP, Lin^−^/Sca1^−^/Kit^+^CD34^−^FCyRII/III^lo/−^) and megakaryocytic progenitors (MkPs, Lin–c-Kit+Sca-1–CD150 + CD41+) (Fig. 1Bi–iii;) relied on a decrease of apoptosis (Fig. 1Biv) within BM cavity. To assess the impact of *Bcor* loss on survival, we monitored our mice cohort for an extended period (18 months). Although Kaplan-Meier analyses showed low survival of *Bcor* null mice (Fig. 1Ci), post-mortem pathological examinations did not reveal any leukemia infiltration in hematopoietic organs (BM, liver, spleen) (Fig. 1Cii). Taken together, these data confirmed that *Bcor* loss negatively influences mice survival by specifically subverting normal hematopoietic compartments and PB output. The absence of a frank acute leukemia phenotype clearly suggested the need of additional cooperative leukemogenic events.

### *Bcor* and *Dnmt3a* loss induces a highly penetrant acute erythroid leukemia (AEL)

We crossed *Bcor* (*Bcor*^*−/−*^) and *Dnmt3a* (*Dnmt3*^*−/−*^) conditional knockout mice with *Mx1-Cre* transgenic mutant to generate *Bcor*^*−/−*^*Dnmt3a*^*−/−*^double knockout mice, single ko *Bcor*^*−/−*^and *Dnmt3*^*−/−*^ mice expressing *Mx1-Cre* and mice with untargeted genes as wild type controls.

**Fig. 2:**
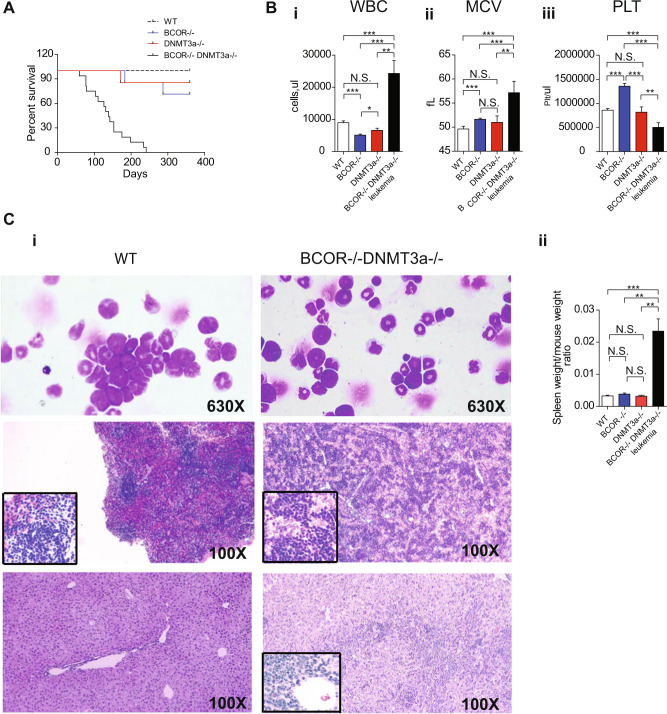
*Bcor* and *Dnmt3a* loss induces a fully penetrant Acute Erythroid Leukemia (AEL). **A** Kaplan–Mayer plot of mice survival according to the indicated genotypes (*n* = 8 to 24 per genotype) *Bcor*^*−/−*^*Dnmt3a*^*−/−*^ mice display a median survival of 135 days (p < 0.0001, Logrank Test). **B** WBCs number (i) and MCV (ii) values in *Bcor*^*−/−*^ *Dnmt3a*^*−/−*^ leukemic mice compared to other control. (iii) Platelets number in *Bcor*^*−/−*^, *Dnmt3a*^*−/−*^, *Bcor*^*−/−*^*Dnmt3a*^*−/−*^ and WT in PB of 3/4 months old mice. **C** i BM cytospin (top panel) hematossilin and eosin of the spleen (middle panel) and liver (bottom panel) in *Bcor*^*−/−*^, *Dnmt3a*^*−/−*^ and WT control mice, showing the blasts infiltration. ×600 of magnification. ii Spleen weight to total body weight ratio in the indicated genotypes. Spleen ratio in *Bcor*^*−/−*^*Dnmt3a*^*−/−*^ (*n* = 9) mice was two fold greater than in *Dnmt3a*^*−/−*^(*n* = 4), *Bcor*^*−/−*^ (*n* = 6) and WT control (*n* = 8) (0.03378 ± 0.0077 and 0.0025 ± 0.000022 vs 0.0042 ± 0.00036 and 0.0035 ± 0.0026 *p* < 0.001 by one-way ANOVA analysis). *p < 0.05, **p < 0.01; ***p < 0.001 unpaired *t*-test with Welch’s correction.

*Bcor*^*−/−*^*Dnmt3a*^*−/−*^ mice developed a fully penetrant and lethal leukemic phenotype with a median survival of 135 days (range from 59 to 234 days), significantly shorter than the other groups (Fig. 2A). Interestingly, we found a *Bcor* deletion both in the single knockout and in the leukemic double *Bcor*^*−/−*^*Dnmt3a*^*−/−*^ knockout mice (Supplementary Fig. 4A), while regarding to *Dnmt3a*, the deletion by Mx1Cre was partial in *Dnmt3a*^*−/−*^ knockout, while its complete loss occurred only in double mutant leukemic mice. Interestingly, in preleukemic mice we detected a strong but not the complete loss of Dnmt3a expression. This suggests that the *Bcor* absence may influence the loss of the *Dnmt3a* gene (Supplementary Fig. 4B, C).

The leukemia diagnosis was primarily based on the presence of leukocytosis and marked macrocytic anemia (Fig. 2Bi, ii). Moreover, the compound mutants showed a consistent drop in platelets number (about 50%) comparing to the preleukemic phase. Conversely, in the other genotypes, platelet numbers remained stable during the entire follow-up period (Fig. 2Biii).

**Fig. 3:**
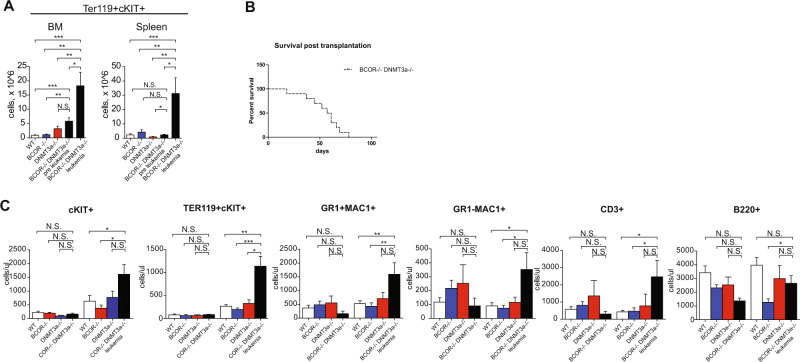
Bcor^*−/−*^Dnmt3a^*−/−*^*shows a* progressive expansion of immature erytrhoid cells populations. **A** TER119 + cKIT+ number of cells in BM (left) and spleen (right) in *Bcor*^*−/−*^*Dnmt3a*^*−/−*^ leukemic mice and preleukemic compared to other control of 3/4 months old mice (*n* = 9, 6, 7, 6, 7). **B** Kaplan–Mayer plot of mice transplanted with 3 × 10^6^ *Bcor*^*−/−*^*Dnmt3a*^*−/−*^ leukemic cells *n* = 12. **C** Total number of cKIT+, Ter119+cKIT+, GR1 + MAC1+, MAC1 + GR−, CD3 and B220+, pre PipC and 3 months post PipC induction in PB of *Bcor*^*−/−*^*Dnmt3a*^*−/−*^, *Bcor*^*−/−*^, *Dnmt3a*^*−/−*^ and WT control (pre *n* = 8,4,4,7; post *n* = 14,8,7,18). *p < 0.05, **p < 0.01; ***p < 0.001 unpaired *t*-test with Welch’s correction.

Consistently with the diagnosis of acute leukemia, BM, PB, and spleen cytospins showed an expansion of nucleated cells with blastic appearance (Fig. 2Ci, top panel, Supplementary Fig. 5ai, ii). The latter was also supported by the presence of paraspinal masses (Supplementary Fig. 5Bi, ii). Disease aggressiveness was confirmed by multiple organ infiltration (including lung, liver, and spleen) and the monotonous blastic populations that partially subverted the local tissue architecture (Fig. 2Ci, ii, middle panel/bottom panel and Supplementary Fig. 5Ci, ii). Flow cytometric analysis demonstrated that leukemic cells co-expressed Ter119 and c-Kit (Fig. 3A), clearly indicating they belonged to the erythroid cell lineage and supporting the diagnosis of acute erythroid leukemia (AEL).

This AEL phenotype was transplantable up to 9 secondary recipients, which developed a lethal AEL with a median survival of 59 days (range 18–78 days) (Fig. 3B). These secondary recipients displayed similar phenotypic characteristics of the primary tumor. Furthermore, the self-renewal capability of the *Bcor*^*−/−*^*/Dnmt3a*^*−/−*^ BM cells was evaluated in replating experiments using colony-forming unit assay (CFU) demonstrating that double knockout cells can regenerate for long time period as compared to other groups (Supplementary Fig. 6).

### *Bcor* and *Dnmt3a* loss induces a displacement towards the erythroid profile starting from early leukemia stages

To better understand the cellular effects of *Bcor* and *Dnmt3a* loss in vivo, we analysed PB and BM samples of unmutated, single and double knockout mice at both early and overt leukemia stages.

**Fig. 4:**
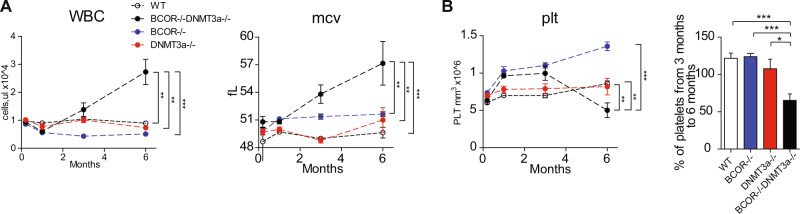
*Bcor*^*−/−*^*Dnmt3a*^*−/−*^ mice develop thrombocytopenia at leukemic phase. **A** Changes in WBC counts (left) and MCV (right) of *Bcor*^*−/−*^*Dnmt3a*^*−/−*^, *Bcor*^*−/−*^, *Dnmt3a*^*−/−*^and WT control during six months. **B** Changes in platelets number (left) and Platelets Ratio between 3 and 6 months (right) of *Bcor*^*−/−*^*Dnmt3a*^*−/−*^, *Bcor*^*−/−*^, *Dnmt3a*^*−/−*^, and WT control during 6 months. *p < 0.05, **p < 0.01; ***p < 0.001 unpaired *t*-test with Welch’s correction.

At leukemia onset, it was observed an expansion of white blood cells (WBC) in *Bcor*^*−/−*^*Dnmt3a*^*−/−*^ due to increased numbers of Gr1 + Mac1+ granulocytes, Gr1+Mac- monocytes and CD3 + lymphocytes together with a progressive expansion of a population of immature cells co-expressing c-Kit and the erythroid marker Ter119 (Fig. 3C). Double knockout mice exhibited a constant increase of WBC count and a drop in the hemoglobin levels (not shown) associated with an increased MCV (Fig. 4A). However, while *Bcor*^*−/−*^ mice showed a progressive increase of platelets counts, the *Bcor*^*−/−*^*Dnmt3a*^*−/−*^ mutant only showed an initial expansion followed by a significant decline associated with leukemic phase (Fig. 4B).

**Fig. 5:**
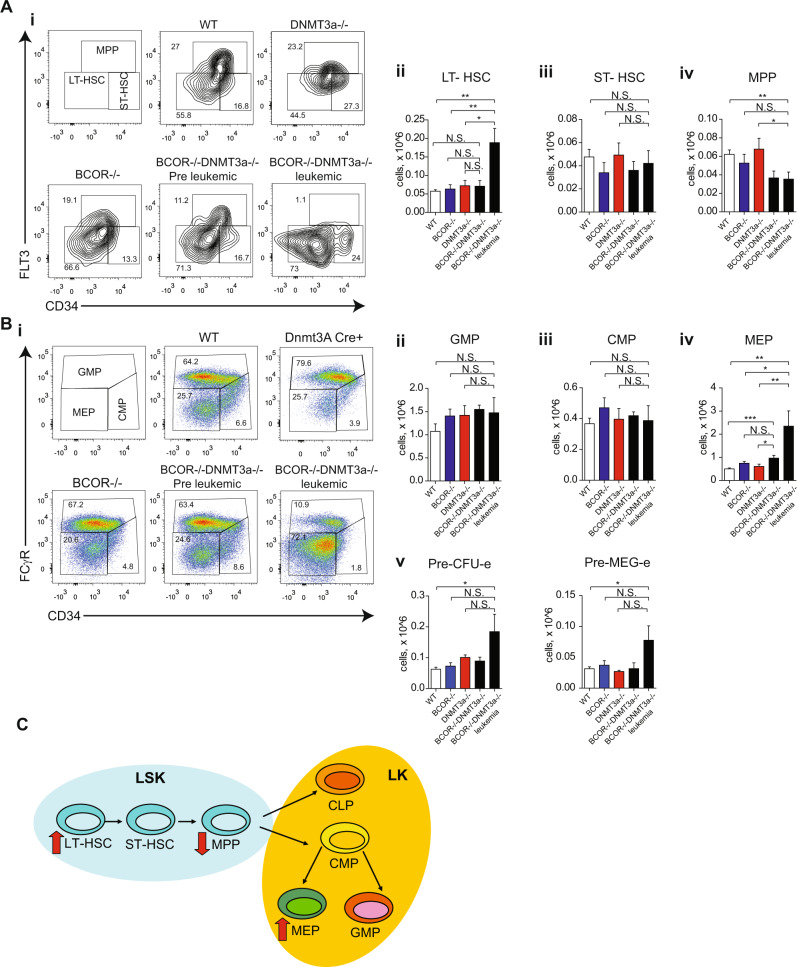
Compound *Bcor* and *Dnmt3a* loss induce a strong displacement toward the erythroid profile in mice. **A** Representative flow-cytometric analysis (i) of stem cell compartment, long term hematopoietic stem cells (LT-HSC Lin^−^/Sca1^+^/Kit^+^CD34l^o/−^FLT3^-^), short term hematopoietic stem cells (ST-HSC Lin^−^/Sca1^+^/Kit^+^CD34^+^FLT3^-^) and multipotent progenitors (MPP Lin^−^/Sca1^+^/Kit^+^CD34^+^FLT3^+^) in BM of *Bcor*^*−/−*^*Dnmt3a*^*−/−*^, *Bcor*^*−/−*^, *Dnmt3a*^*−/−*^and WT mice. Total number (Right panel) of LT-HSC (ii), ST-HSC (iii) and MPP (iv) in BM of *Bcor*^*−/−*^*Dnmt3a*^*−/−*^, *Bcor*^*−/−*^, *Dnmt3a*^*−/−*^and WT mice. **B** Representative flow-cytometric analysis (i) of progenitors cell compartment, granulocyte-macrophage progenitors (GMP Lin^−^/Sca1^−^/Kit^+^CD34^+^FCgRII/III^+^), common-myeloid (CMP Lin^−^/Sca1^−^/Kit^+^CD34^+^FCgRII/III^−^) and megakaryocyte-erythroid progenitors (MEP Lin^−^/Sca1^−^/Kit^+^CD34^−^FCgRII/III^lo/−^) in BM of *Bcor*^*−/−*^*Dnmt3a*^*−/−*^, *Bcor*^*−/−*^, *Dnmt3a*^*−/−*^and WT mice. Total number of GMP (ii), CMP (iii) and MEP (iv) in BM of *Bcor*^*−/−*^*Dnmt3a*^*−/−*^, *Bcor*^*−/−*^, *Dnmt3a*^*−/−*^ and WT mice. (*n* = 16, 12, 10, 9) (v) Total number of (Pre-MegE Lin^−^/FCgRII/III^−^ CD150^+^CD105^−^) and Pre-CFUe (Lin^−^/FCgRII/III^-^ CD150^+^CD105^+^) in BM of *Bcor*^*−/−*^*Dnmt3a*^*−/−*^, *Bcor*^*−/−*^, *Dnmt3a*^*−/−*^ and WT mice. (*n* = 16, 12, 10, 9) **C** Summary of BM hemopoietic development in mice; red arrows indicate the deregulated populations. *p < 0.05, **p < 0.01; ***p < 0.001 unpaired *t*-test with Welch’s correction. .

Cellular alterations of BM and spleen were analysed 2–3 months after activation of conditional mutations in all genotypes to investigate early leukemic stages. For comparison, we included in the analysis *Bcor*^*−/−*^*Dnmt3a*^*−/−*^ mice developing leukemia 4–6 months after pIpC induction of the Cre-recombinase. Lin^−^/Sca1^+^/Kit^+^ (LSK) stem cell compartment examination revealed a significant accumulation of long term hematopoietic stem cells (LT-HSC Lin^−^/Sca1^+^/Kit^+^CD34^lo/−^FLT3^−^) in leukemic *Bcor*^*−/−*^*Dnmt3a*^*−/−*^ BM compared to other groups, a feature not found at early leukemic stages (Fig. 5Ai, ii Supplementary Fig. 7).

Short term hematopoietic stem cells (ST-HSC Lin^−^/Sca1^+^/Kit^+^CD34^+^FLT3^−^) were markedly lower only in *Bcor*^*−/−*^ genotype. Multipotent progenitors (MPP Lin^−^/Sca1^+^/Kit^+^CD34^+^FLT3^+^) were lower in double mutant mice both at early and overt leukemic stages. However, statistical significance was only reached when MPPs numbers were compared to wild type animals (Fig. 5Aiii, iv). The analysis of changes occurring during lineage commitment and maturation revealed a striking 5-fold increase of megakaryocyte-erythroid progenitors (MEP Lin^−^/Sca1^−^/Kit^+^CD34^−^FCgRII/III^lo/−^) in preleukemic and leukemic *Bcor*^*−/−*^*Dnmt3a*^*−/−*^ mice (Fig. 5Bi, iv). Accordingly, only leukemic mice displayed a marked expansion of other more committed erythroid-restricted progenitors including the bipotent pre-megakaryocyte erythrocyte (Pre-MegE Lin^−^/FCgRII/III^−^ CD150^+^CD105^−^) and Pre-CFUe (Lin^−^/FCgRII/III^−^ CD150^+^CD105^+^) (Fig. 5Bv).

Given the erythroid skewing of HSC differentiation of *Bcor*^*−/−*^*Dnmt3a*^*−/−*^ mice, we used CD71 and Ter119 staining to further characterize later downstream stages of RBCs development within the BM. Our data demonstrated an accumulation of proerythroblasts both at preleukemic and leukemic stages, while the increase of early basophilic, late basophilic, chromatophilic, and orthochromatophilic erythroblasts was detected only in double knockout leukemic mice (Supplementary Fig. 8A).

Altogether, these data suggest that the development of overt leukemia is predated by changes in the composition of hematopoietic stem/progenitor cells compartment, more detectable among myeloid and erythroid progenitors (Fig. 5C).

No significant differences emerged in total number of different myeloid committed progenitors including granulocyte-macrophage (GMP Lin^−^/Sca1^−^/Kit^+^CD34^+^FCgRII/III^+^), common-myeloid (CMP Lin^−^/Sca1^−^/Kit^+^CD34^+^FCgRII/III^−^) (Fig. 5Bii, iii) and pre-granulocyte-monocyte progenitors (Pre-GM Lin^−^/FCgRII/III^−^ CD150^−^CD105^−^), as well as more mature BM granulocytes and monocytes, among all littermate groups (data not shown).

### *Bcor* and *Dnmt3a* loss determines altered GATA factor switching and changes of p53 family members in LSK and MEP compartments

Next, we performed RNA-seq experiments to get insight on the molecular changes of *Bcor* and *Dnmt3a* cooperation in AEL development. We performed a meta-analysis of the differentially regulated genes from the following pairwise comparisons: leukemic *Bcor*^*−/−*^*Dnmt3a*^*−/−*^ vs wild type, *Bcor*^*−/−*^ vs wild type, *Dnmt3a*^*−/−*^ vs wild type. Moreover, we searched for significant two-way and three-way overlaps among the differentially expressed gene lists. Analysis was done on both LSK and MEP populations, given the significant expansion of these cells in the BM of leukemic mice.

**Fig. 6:**
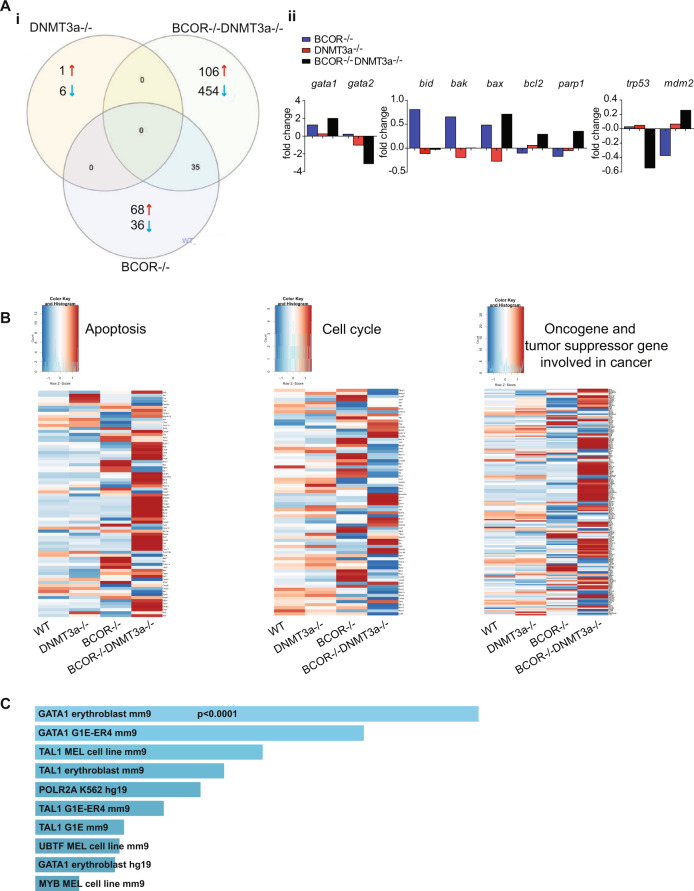
Deregulated genes in *Bcor*^*−/−*^*Dnmt3a*^*−/−*^ leukemia. **A** (i) Overlap of differently gene expression (RNAseq) in the LSK cells of *Bcor*^*−/−*^*Dnmt3a*^*−/−*^, *Bcor*^*−/−*^, *Dnmt3a*^*−/−*^compared to WT control (*n* = 3 mice for each genotype). (ii) mRNA expression in the LSK cells of the indicated genotypes for the most up- and downregulated genes. **B** Heatmaps of apoptosis pathway (left), cell cycle (middle), cancer (right) in LSK cell for the indicated genotype. **C** Enrichr bar plot (https://amp.pharm.mssm.edu/Enrichr/). Bars represent the proportion of genes upregulated in both human AEL samples (*n* = 137) and *Bcor*^*−/−*^*Dnmt3a*^*−/−*^ LSK (*n* = 3) whose promoter is bound by the indicated transcription factor.

*Bcor*^*−/−*^*Dnmt3a*^*−/−*^ LSK and MEP showed a large number of differentially expressed genes (560 and 269, respectively). Within LSK population, 106/560 were upregulated and 454/560 were downregulated (Fig. 6Ai, Table S1) while, within MEP compartment 133/269 were upregulated and 136/269 downregulated (Fig. 6Ai, Supplementary Fig. 9A, Table S2). There were 35 genes for LSK and 17 for MEP transcripts commonly altered between *Bcor*^*−/−*^*Dnmt3a*^*−/−*^ and *Bcor*^*−/−*^, on the other hand, there were no transcripts commonly altered in all pairwise comparisons with *Dnmt3a*^*−/−*^ (Fig. 6Ai and Supplementary Fig. 9A).

Interestingly, GATA1 scored as one of the most upregulated genes in LSK compartment in line with an aberrant erythroid skewing originating in the hematopoietic compartment of leukemic mice. In these cells, GATA2 levels were highly downregulated (Fig. 6Aii) proving further evidence of an altered switching of GATA factors as a driving event in *Bcor*^*−/−*^*Dnmt3a*^*−/−*^ mice with AEL (Table S1). The dysregulation on GATA family members was not detected in MEP compartment of leukemic mice, confirming that this defect was directly linked to the leukemic stem cell population (Table S2, Supplementary Fig. 9B).

Additional pathway analysis of LSK and MEP cells from *Bcor*^*−/−*^*Dnmt3a*^*−/−*^ mice showed the enrichment of deregulated genes involved in cellular apoptosis, such as BID and BAK pro-apoptotic genes and the anti-apoptotic Bcl2 family genes. The PARP1 pro-survival gene was strongly upregulated in LSK only (Fig. 6Aii and Supplementary Fig. 9B, C). In addition, we found a notable decline of p53 levels, that has been driven by the marked elevation of MDM2, that is its master antagonist in the cell cycle machinery [28] (Fig. 6Aii, b and Supplementary Fig. 9B, C).

In order to search for shared molecular alterations, RNA-seq data on mouse LSK cells were used as a backbone for comparison with human AEL samples [29]. By interpolating these data with the list of deregulated genes in our leukemic mice, we found 104 genes, commonly upregulated, and 136 genes, commonly downregulated, between mouse LSK cells and human AEL (Supplementary Fig. 10A, B, Supplementary Fig. 11, Table 1). Within genes upregulated in both human and mouse cells, there was a striking enrichment of genes regulated by GATA1 (Fig. 6C), suggesting a pivotal role of this transcription factor in driving AEL. These findings provided evidence that our AEL mouse may serve as an operational platform for human *Bcor*^*−/−*^*Dnmt3a*^*−/−*^ leukemia modeling.

**Table 1:** List of the commonly altered genes, up (right) and down (left) regulated between BCOR^*−/−*^DNMT3a^*−/−*^ LSK mouse cells and Human AEL.

Human- Mouse			
UP		DOWN	
ABCB9	MOSPD1	ABHD14A	MFGE8
ACAP2	MXI1	ACCS	MICU1
ACBD3	MYO5B	ALDOC	MOB3A
ACHE	NCKAP1	ANKRD46	MOCS2
ACSL1	NDUFS1	ANXA4	MORN4
ACSL6	NOM1	APOM	MRE11A
AFF2	OAT	APOO	NIT2
ARF6	OSTM1	ARMCX4	NRM
ARFGEF1	PDE12	ATG10	NT5C3B
ARG1	PDZD8	AUTS2	NUDT14
ASAP1	PFKFB3	BDH2	OSBPL1A
ASNS	PHF10	BEX2	OXA1L
BAIAP3	PLEKHF2	BIVM	P2RY14
BAZ1A	POLG	CAPNS1	PAPSS1
BCL6	POMC	CCDC28A	PEMT
BLOC1S4	PPM1D	CENPM	PEX11G
BRAF	RAB22A	CENPT	PGK1
CAB39	RANBP2	CPXM1	PHF19
CCNY	REPS2	CTC1	PHF20
CEP76	RFXAP	CTR9	PIGL
CHD7	RGCC	DHRS4	POC5
CHFR	RHOB	DHX30	POR
CLPTM1L	RIN1	DNMT3A	PPFIBP1
CRAT	RSBN1	ECHDC2	PPP2R1A
DCUN1D5	SCYL2	ECSCR	PRMT1
DDHD1	SLC16A1	ELK3	PSPH
DLEU2	SLC25A21	EMCN	PSRC1
DNAJC25	SMC4	ENO1	RANBP17
DRP2	SPATA18	EPHB4	RBMX
EME2	SSX2IP	ERI3	REC8
EXOC5	STK11	FABP5	RPAP2
FAM20B	STT3B	FAM64A	SASH3
FBXW2	TACC1	FAM69B	SELP
GARS	TADA2B	FBXL2	SGCB
GLRX5	TARS	FBXO16	SIRT3
GPC4	TMEM167B	FBXO6	SIRT5
HCFC2	TMEM56	FTO	SLC2A9
HDLBP	TMEM9B	FXYD5	SOD1
HIPK3	TOP1	GALNT11	SORBS3
HIST1H4I	TRIP12	GAS8	SORD
HIST4H4	UBA5	GCAT	SPAG16
IBA57	UBE2Q1	GEMIN8	SSBP2
KLF1	UBE2S	GOT2	STAP1
MAN2A1	UBXN2A	GSTK1	SV2A
MAP2K4	UFSP1	GTF2IRD1	TBC1D16
MAPKAPK5	UGGT1	HNRNPA1	TBXAS1
MCPH1	UROS	HSCB	TCEAL1
METAP2	USP15	HYAL3	TERT
MFSD2B	USP33	IFITM3	TEX9
MINPP1	WAPAL	IFT122	TLDC1
MMP14	WDR7	IL12RB2	TMEM231
MON2	ZDHHC5	INCA1	TMEM41A
		IRAK1BP1	TMEM98
		ITGA2	TOX
		JAM2	TPM2
		JKAMP	TRMT61B
		JPX	TSPAN3
		KHDRBS3	TSTD2
		KLHL3	TTC7B
		LETMD1	TXLNA
		LGALS3BP	TYW3
		LRRC36	UBE2I
		LRRC49	UBE3B
		LUC7L	UNG
		LYRM9	YY2
		MAGED2	ZCCHC10
		MBOAT4	ZIK1
		METTL10	ZSCAN2

### Decitabine exerts inhibitory effects on the *Bcor/Dnmt3a* null leukemic mice

In order to assess the impact of anti-leukemic therapies on *Bcor*^*−/−*^*Dnmt3a*^*−/−*^AEL mice, we tested the therapeutic efficacy of the standard chemotherapeutic agent cytarabine compared to the demethylating agent decitabine in lethally irradiated CD45.1 recipient mice transplanted with leukemic cells. The rationale for using decitabine was based on the fact that the *Bcor* transcriptional repression activity is mediated by histone demethylase and the function of *Dnmt3a* is to catalyse the addition of methyl groups to CpG dinucleotides. Thus, in our double knockout mice, we expected a hypomethylation status with leukemogenic potentials.

**Fig. 7:**
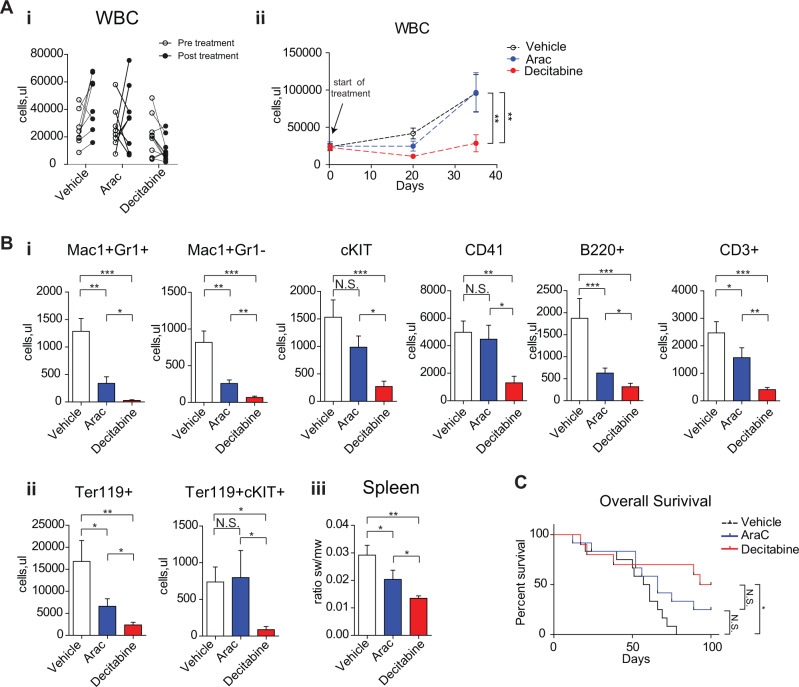
Decytabine exert inhibitory effects on the *Bcor*^*−/−*^*Dnmt3a*^*−/−*^ leukemic population. **A** WBC count changes (i) in leukemic *Bcor*^*−/−*^*Dnmt3a*^*−/−*^ mice pre and 3 days after the end of the treatment with decytabine (DEC), cytarabine (ARAC) and vehicle (VEI). (ii) WBC follow up (right) during and post the treatment with DEC, ARAC and VEI in leukemic mice. **B** (i) Total number of GR1 + MAC1 + , MAC1 + GR1-, cKIT+, CD41+, B220+ and CD3+ cells in PB 3 days after DEC, ARAC and vehicle treatment in leukemic *Bcor*^*−/−*^*Dnmt3a*^*−/−*^, mice (*n* = 11,11,12). (ii) Total number of TER119+ and TER119 + /cKIT+ cells in PB 3 days after DEC, ARAC and vehicle treatment in leukemic *Bcor*^*−/−*^*Dnmt3a*^*−/−*^ mice (*n* = 11,11,12). (iii) Spleen weight to total body weight ratio in *Bcor*^*−/−*^*Dnmt3a*^*−/−*^ leukemic mice after each indicated treatment (*N* = 4,4,3). **C** Kaplan–Mayer plot of leukemic *Bcor*^*−/−*^*Dnmt3a*^*−/−*^ mice survival after DEC, ARAC and VEI treatment to the indicated genotypes (*n* = 10 to 9 per genotype) (*p* < 0.0001, Logrank Test). *p < 0.05, **p < 0.01; ***p < 0.001 Wilcoxon matched pairs test.

Mouse treatments started at leukemia onset, defined by the presence of WBC count above 20,000 cells/μl and/or high MCV values and/or low platelets number. Cytarabine was administered for 7 days continuously, while decitabine for a total of 5 administrations twice a week, followed by the assessment of disease burden (Fig. 7Ai). WBC count was significantly reduced at the end of decitabine treatment compared to vehicle, while chemotherapy determined only a modest impact on leukocytosis (Fig. 7Aii). Two weeks after the end of treatments, WBC count was significantly lower in decitabine group, compared to cytarabine and vehicle ones. PB flow cytometry showed a significant reduction of immature c-KIT and Ter119 + c-KIT + cells after decitabine compared to other treatments (Fig. 7Bi, ii), as also confirmed by post-mortem examination on pathological splenic specimens (Fig. 7Biii), thus indicating that *Bcor*^*−/−*^*Dnmt3a*^*−/−*^ leukemic cells were more sensitive to hypomethylating agent than to chemotherapy. Moreover, there was a tendency for decitabine treated mice toward the achievement of a longer survival, compared to cytarabine and vehicle groups (Fig. 7C).

## Discussion

Somatic recurrent loss-of-function mutations of *Bcor* have been detected in AML [7] but their precise role in normal and malignant hematopoiesis is still under investigation. Here, we demonstrate for the first time that *Bcor* deficiency perturbs erythro*-*megakaryopoiesis and cooperates with *Dnmt3a* loss (a phenotype partially recapitulating that of heterozygous *Dnmt3a*^R662H^ mutations [30]) in promoting AEL in mice.

Perturbation of the erythroid compartment in our *Bcor*-deficient mice was characterized by a decrease in the total number of red blood cells and macrocytosis, confirming previous observations from Tara et al. [19] who demonstrated mild macrocytic anemia in mice after the deletion of *Bcor* exons 9 and 10. Besides the negative effects on erythroid cells, we also found a strong increase in platelets counts due to a significant expansion of the MKP population in BM.

Contribution of *Bcor* loss in promoting megakaryocytic proliferation in mice is a novel finding that is also of potential clinical relevance. A missense *Bcor* mutation has been reported in a patient with triple-negative essential thrombocytemia and normal karyotype, suggesting a possible role of this variant in the pathogenesis of the disease [31]. Moreover, *Bcor* mutations may occur in myelodysplastic/myeloproliferative neoplasms (MDS/MPNs) that share dysplastic and proliferative features [32]. MDS/MPN disorders include chronic myelomonocytic leukemia (CMML) and MDS/MPN with ring sideroblasts and thrombocytosis (MDS/MPN-RS-T) in which *Bcor* is recurrently mutated in 7.4% [33] and 24% of cases [34], respectively. Although the most frequent mutation in MDS/MPN-RS-T is that affecting the *SF3B1* gene [35, 36], the presence of variants in epigenetic genes, such as *Bcor*, may contribute to the development of this myeloid neoplasm. Co-occurrence of erythroid blood cell alterations and thrombocytosis in our conditional knockout *Bcor* mice support this hypothesis.

Similar to previous models [17, 19], *Bcor* deficiency alone was not sufficient to drive a myeloid malignancy in our mice. However, we could demonstrate that the combined *Bcor* and *Dnmt3a* loss promoted *a* fully penetrant AEL phenotype that killed mice in 5–6 months. Leukemic mice showed leukocytosis due to the expansion of c-Kit+ blasts expressing the erythroid marker Ter119, macrocytic anemia, and progressive reduction of the thrombocytosis driven by the single *Bcor* deletion. The analysis of BM subpopulations showed an erythroid skewing of HSC differentiation demonstrating that AEL was driven by loss of *Bcor* and *Dnmt3a*.

The 2016 WHO classification of hematopoietic tumors has adopted rather restrictive criteria for the diagnosis of pure erythroid leukemia (or AEL), moving cases with an increased percentage of myeloid blasts (previously named erythroleukemia) in the group of MDS [37–39]. Thus AEL, described for the first time by Di Guglielmo in 1928 under the term of “*acute erythremic myelosis*” [40], is now included as an entity within the category of AML not otherwise specified (NOS) of WHO-2016 [37]. However, targeted next-generation sequencing has clearly demonstrated the molecular heterogeneity of this apparently homogeneous morphological form of AML [41, 42] that shows a mutational spectrum intermediate between MDS and AML [29]. Notably, two cases of AEL were characterized by the co-occurrence of *Bcor* and *Dnmt3a* mutations only [29], thus supporting the findings described in our mice model.

In order to define the gene expression signature of AEL in our mice, we performed RNAseq analysis on the LSK population of the four different genotypes. We identified an altered switching of the Gata factors that are known to play a key role in controlling mechanisms underlying erythroid differentiation [43]. Specifically, *Gata1* was one of the most upregulated genes in leukemic mice compared to the other genotypes whereas *Gata2* was downregulated. The latter finding is unexpected since in physiological conditions [44], the transcription factor Gata2 is highly expressed in hematopoietic stem cells, whereas its expression declines after erythroid commitment of progenitors [43]. In contrast, the start of Gata1 expression coincides with the erythroid commitment and increases along with the erythroid differentiation. Collectively, these data provide evidence for an imbalance towards an erythroid phenotype starting from the LSK stage and point to Gata factors deregulation as an early event altering the HSC fate and sensitizing cells to further malignant transformation in the context of concurrent *Bcor* and *Dnmt3a* deficiency, as it has been also very recently demonstrated in both human and mouse AEL samples [45].

Our AEL mouse model also exhibited dysregulation of various oncogenes and tumor suppressor genes involved in cancer pathways, including decline of Tp53 levels and marked elevation of Mdm2. Accordingly, an in vivo mouse model demonstrated a key driver leukemogenic function of *Tp53* supported by the potent interaction with *Ntrk1* [29]. Moreover, the presence of more than a single *TP53* abnormality seems to play a key role in the molecular pathogenesis of AEL in patients [46].

AEL is usually poorly responsive to intensive chemotherapy and shows a dismal outcome [47] that may be due, at least in part, to the accumulative impaired TP53 function and consequent genomic instability. Similarly, our AEL mice were resistant to the nucleoside analog cytarabine but showed significantly improved survival when treated with the epigenetic drug decitabine. The benefit of this drug suggests an important role of epigenetics in promoting AEL in our *Bcor*^*−/−*^*Dnmt3a*^*−/−*^ compound mice. Accordingly, the presence of *TP53* mutations appears to be associated with a high degree of decitabine sensitivity in AML patients in one study [48], although it has not been confirmed in another study [46].

In conclusion, we provide the first demonstration that concurrent *Bcor* and *Dnmt3a* loss promotes AEL in mice and sheds light, at least in part, on the cellular and molecular features underlying this leukemia. Our model also represents a potential platform for the identification and validation of drugs for improving therapy of AEL patients.

## Supplementary information

Supplementary Material
