# Supplementary material for: Bcor deficiency perturbs erythro-megakaryopoiesis and cooperates with Dnmt3a loss in acute erythroid leukemia onset in mice
Source: Leukemia. 2020 Nov 6;35(7):1949–63. doi: 10.1038/s41375-020-01075-3 (PMC8257496; doi:10.1038/s41375-020-01075-3)
Supplement: Supplementary file 1 — Supplementary Material [file 41375_2020_1075_MOESM1_ESM.pdf]

SUPPLEMENTARY METHODS

Mouse strains

To generate compound *bcor/DNMT3a* (background C57Bl/6) mutant animals, we crossed Knock-out *Bcor (*background C57Bl/6) with *DNMT3a* Knock-out mice (background C57Bl/6) and used the Mx1Cre transgenic line to induce the transgenic activation in hematopoietic stem cells.

Expression of Mx1-Cre and excision of PGK-Neo cassette were induced by polyinosinic-polycytidylic acid (pIpC) treatment in vivo: 8- to 12-week-old mice were injected intraperitoneally with 250 mg/dose pIpC (Sigma-Aldrich, St. Louis, MO) every other day for 3 injections. Mice were analyzed starting 2 months after pIpC induction.

In vitro hematopoietic colony-forming assay

For in vitro replating assays, a total of $15x10^3$ BM cells were plated in duplicate in MethoCult M3434 (StemCell Technologies) containing rm SCF, rm IL-3, rh IL-6, rh EPO and grown at 37°C with 5% CO2. All colonies were quantified after 10-12 days of growth for detection of BFU-E, CFU-GM, CFU-GEMM in BM.

Transplant

Congenic Ly.5.1 mice were Lethally irradiated (9 Gy) and transplanted with 2x10^6 BM cells derived from *Bcor/DNMT3a* leukemic mice as donor cells and 0.5x10^6 BM cells from Ly5.1 healty mice as helper.

Flow Cytometry and Cell Sorting antibodies and methods

For FACS analysis and cell sorting we used the following eBioscience (Thermo-Fisher scientific) antibodies: FITC-anti-Gr1 (11-5931-85), PE-anti-Mac1 (12-0112-85), APC-anti-cKit (17-1171-83), AlexaFluor780-anti-B220 (47-0452-82), PercP5.5-anti-CD3 (35-0031-82), FITC-anti-Ter119 (11-5921-85), PE-anti-CD41 (12-0411-83), APC-anti-CD71 (17-0711-82), AlexaFluor780-anti-cKIt (47-1171-82); BM cells were also stained with PercP5.5-conjugated antibodies against CD3 (35-0031-82), CD4 (45-0042-82), CD8 (45-0081-82), B220 (45-0452-82), Ter119 (45-5921-82), Mac-1 (45-0112-82), Gr-1 (45-5931-80), IL7R (45-1271-82), CD5 (45-0051-82), allophycocyanineFluor-780–conjugated anti–c-Kit (47-1171-82), FITC-conjugated anti-CD41 (11-0411-85), allophycocyanin-conjugated

anti-FCgR (17-0161-81), PE/CY7-conjugated anti-CD105 (120410, BioLegend), PE-conjugated anti-CD150 (115904, BioLegend), FITC-anti-CD34 (11-0341-85), PE-anti-CD135 (12-1351-83) and PE/CY7-anti-Sca1 (25-5981-82). Multiparameter flow cytometry was used to define and analyze the HSC compartment, including long-term hematopoietic stem cells (LT-HSC; lin-Sca-1+c-kit+ CD34−Flt3−), short-term HSC (ST-HSC; lin-Sca-1+c-kit+CD34+Flt3−) and multipotent progenitors (MPP; lin-Sca-1+c-kit+CD34+Flt3+). In addition, the myeloid progenitors population was included and analyzed as common myeloid progenitors (CMPs Lin−Sca-1−cKit+CD34+FcγRII/IIIlo), granulocyte/monocyte progenitors (GMP Lin−Sca-1−cKit+CD34+FcγRII/IIIhi) and common megakaryocyte-erythroid progenitor (MEP Lin−Sca-1−cKit+CD34−FcγRII/IIIlo) populations.

Real time-PCR

mRNA from sorted LSK cells was extracted with mRNeasy micro kit (Qiagen), retrotrascribed to cDNA using the Superscript IV First-Strand Synthesis System (Invitrogen) and DNMT3a levels were evaluated by qPCR (Applied Biosystem 7300 Real time PCR system). The following primers for Dnmt3a Ex19 F: TTTGAGTTCTACCGCCTCCT and Dnmt3a Ex20 R: GTGTGCAGCAGACACTTCTT were used. Beta-Actin was used for normalization and ΔΔCt formula used to calculate mRNA fold change.

Gene expression profiling human/mice

For the comparison between humans and mice, data from patients of AEL were taken into consideration, selecting patients with genotypes similar to those of the mouse and comparing them with healthy controls[47]. (Supp.Fig11) Starting from the CPM values, the differential analysis was performed using a T-test between AEL patients and controls and a Fold Change was calculated considering the difference of logarithms based on the averages of the two populations. The results were filtered by p-value (0.05), so that they could be compared with those identified in the mouse and filtered in the same way.

Statistical analysis

For survival studies, we used the method of Kaplan and Meier with a follow-up of 20 mice per genotype. For studies in which animals are sacrificed for analysis at different ages, we analyzed the data using “analysis of variance”. For *in vitro* assays, we utilized a standard

Students t-test. Statistical analysis was carried out on at least three independent experiments.

Supplementary figure legends:

Supplementary Figure1: A) Schematic representation of the recombined allele screening strategy. **B)** Southern blot analysis on ES cells, showing the omologous recombination occurred. **C) (i)** PCR genotyping strategy for the mutated *Bcor* allele and WT from a DNA WT, heterozygous *Bcor*$^{+/-}$ and homozygous *Bcor*$^{-/-}$ mice. WT fragment 301 bp *Bcor* recombination 393 bp. **(ii)** Top, PCR strategy showing the occurred recombination in *Bcor* locus in the BM; middle, PCR strategy identifying CRE+ and CRE- (WT) mice. 200 bp. Bottom, PCR strategy showing the mutated *Bcor* allele (393 bp) and WT (301 bp) in the BM of WT, heterozygous *Bcor*$^{+/-}$ and homozygous *Bcor*$^{-/-}$ mice.

Supplementary Figure2: A) (i) *Bcor* mRNA sequence in WT and *Bcor* knock-out mice. **(ii)** Western blot analysis of *Bcor* expression in BM of *Bcor*$^{-/-}$ and *Bcor*$^{+/+}$ mice and positive control OCI/AML3.* p<0.05, unpaired t-test with Welch's correction

Supplementary Figure3: A) Leukopenia was mainly attributed to reduced numbers of B lymphocytes. **(i)** WBC changes in 12 months in PB of *Bcor*$^{-/-}$, *Bcor*$^{+/-}$ and WT mice. White blood cells (WBC) **(ii)**, B220+ **(iii)**, GR1+MAC1+ and CD3+ **(iv)** number of cells in PB of *Bcor*$^{-/-}$, *Bcor*$^{+/-}$ and WT from 6-12 months old mice. **(v)** Total number of B220+ cells in BM of *Bcor*$^{-/-}$, *Bcor*$^{+/-}$ and WT mice. **B)** PLT changes in 12 months in PB OF of *Bcor*$^{-/-}$, *Bcor*$^{+/-}$ and WT mice. **C)** PLT number **(i)** and ratio between basal (pre PIPC induction) and time point **(ii)** in in PB of *Bcor*$^{-/-}$, *Bcor*$^{+/-}$ and WT mice. * p<0.05, ** p<0.01 *** p<0.001; unpaired t-test with Welch's correction.

Supplementary Figure4: A) Schematic representation of *Bcor* mRNA sequence in wild type, *Bcor*$^{-/-}$, *Dnmt3a*$^{-/-}$ and *Bcor*$^{-/-}$*Dnmt3a*$^{-/-}$ mice. **B)** Schematic representation of *Dnmt3a* mRNA sequence in wild type, *Bcor*$^{-/-}$, *Dnmt3a*$^{-/-}$ and *Bcor*$^{-/-}$*Dnmt3a*$^{-/-}$ mice. **C)** Real time-PCR reveals reduction of *Dnmt3a* mRNA levels in *Bcor*$^{-/-}$*Dnmt3a*$^{-/-}$ pre-leukemic LSK cells compared to WT.

Supplementary Figure5: A) (i) PB smear showing the blasts presence in leukemic *Bcor*$^{-/-}$*Dnmt3a*$^{-/-}$ mice compared to WT. **(ii)** Spleen cytospin in leukemic *Bcor*$^{-/-}$*Dnmt3a*$^{-/-}$ and WT control mice, showing the blasts presence. 400X of magnification. **B)** Hematoxylin and eosin in Spinal bone marrow in leukemic *Bcor*$^{-/-}$*Dnmt3a*$^{-/-}$ and WT control mice, showing the blasts infiltration 100X **(i)** and 400X **(ii)** of magnification. **C) (i)** Hematoxylin and eosin

in lung of leukemic *Bcor$^{-/-}$Dnmt3a$^{-/-}$* and WT control mice, showing the blasts presence. 100X of magnification and 400X **(ii)**.

Supplementary Figure6: A-B) Time course of colony forming unit (A) and total number of cells (B) from mouse BM cells, plated in equal number for each genotype (0,15x10^5) in semi solid stem cells methocult. CFU were counted every 14 days.

Supplementary Figure7: Total number of LSK (left panel) ($Lin^{-}/Sca1^{+}/Kit^{+}$) and LK (right panel) ($Lin^{-}/Sca1^{+}/Kit^{-}$) in BM of *Bcor$^{-/-}$Dnmt3a$^{-/-}$*, *Bcor$^{-/-}$*, *Dnmt3a$^{-/-}$* and WT mice (n= 16, 12, 10, 9). * p<0.05, ** p<0.01; *** p< 0.001 unpaired t-test with Welch's correction.

Supplementary Figure8: A) Flow cytometry (left) and total number (right) of $Ter119^{med}CD71^{hi}$ (pro Erythroblast), $Ter119^{hi}CD71^{hi}$ (Early Basophilic), $Ter119^{hi}CD71^{med}$ (Late Basophilic) and $Ter119^{hi}CD71^{low}$ (Chromatophilic and Ortochromatophilic) represented as I, II, III, and IV, respectively in BM of *Bcor$^{-/-}$Dnmt3a$^{-/-}$* (pre Leukemic and leukemic), *Bcor$^{-/-}$, Dnmt3a$^{-/-}$* and WT. * p<0.05, **p<0.01, *** p<0.001; unpaired t-test with Welch's correction

SupplementaryFigure9: (A) Overlap of differently gene expression (RNA seq) in the MEP cells of *Bcor$^{-/-}$Dnmt3a$^{-/-}$*, *Dnmt3a$^{-/-}$* and *Bcor$^{-/-}$* compared to WT control (n=3 mice for each genotype). **B)** mRNA expression in the MEP cells of the indicated genotypes for the most up and down regulated genes **C)** Heatmaps of Apoptosis pathway (left), cell cycle (middle left), Cancer (middle rght) and Oxidative phosphorylation (right) in MEP cell for the indicated genotype.

SupplementaryFigure10: A-B) Overlap of the differently gene expressed, up regulated **(A)** and down regulated **(B)** in the human AEL (n=137) and mouse *Bcor$^{-/-}$Dnmt3a$^{-/-}$* (n=3) LSK cells. 137 Human AEL were compared with 7 healthy patients, while *Bcor$^{-/-}$Dnmt3a$^{-/-}$* (n=3) LSK cells were compare with LSK cells derived from 3 WT control mice.

SupplementaryFigure11: A) The samples were analyzed by PCA to establish the level of clustering and, as a result, three samples were excluded from AEL patients and one from controls to obtain two populations as homogeneous as possible.

.

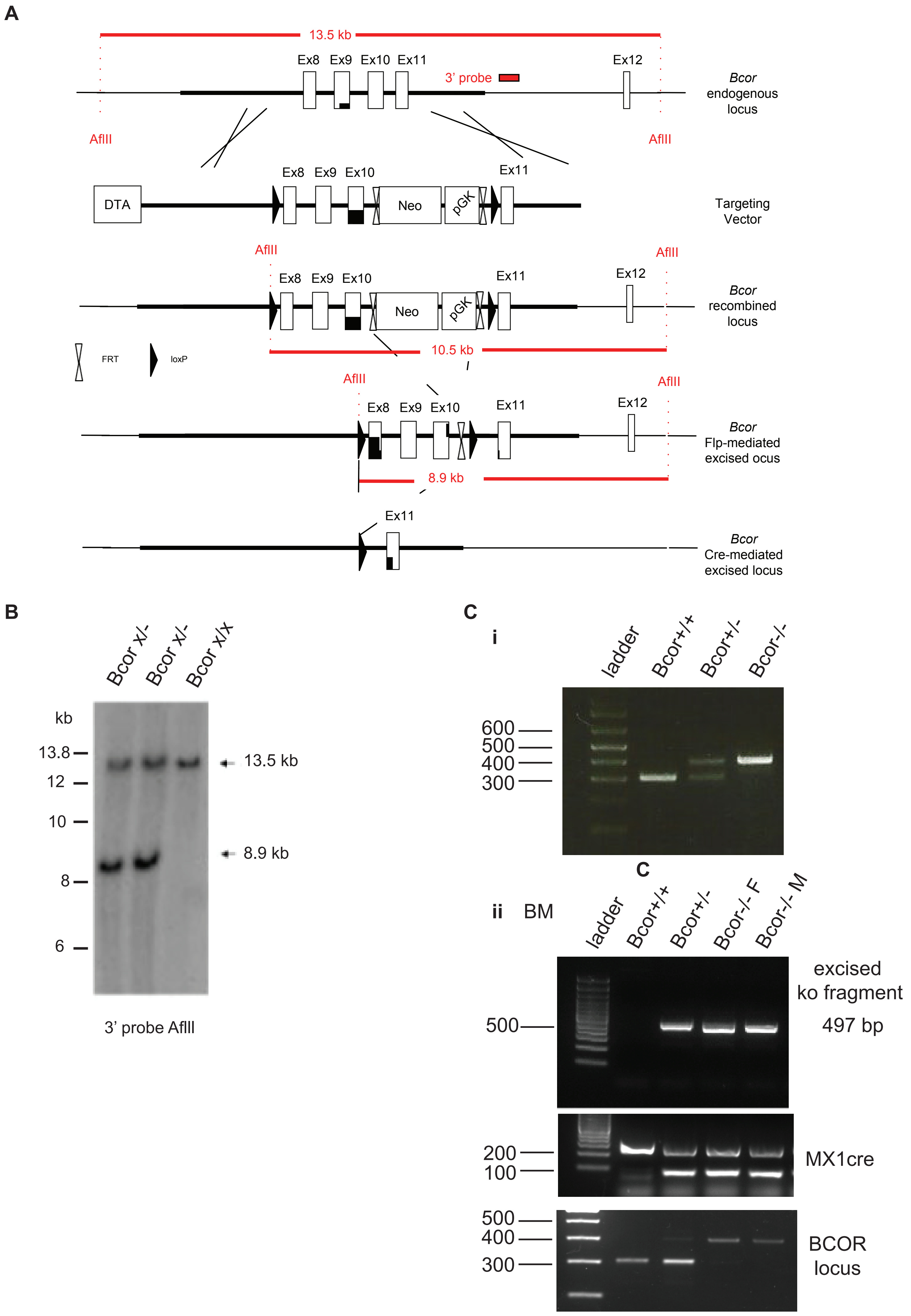

Supplementary Figure 1.

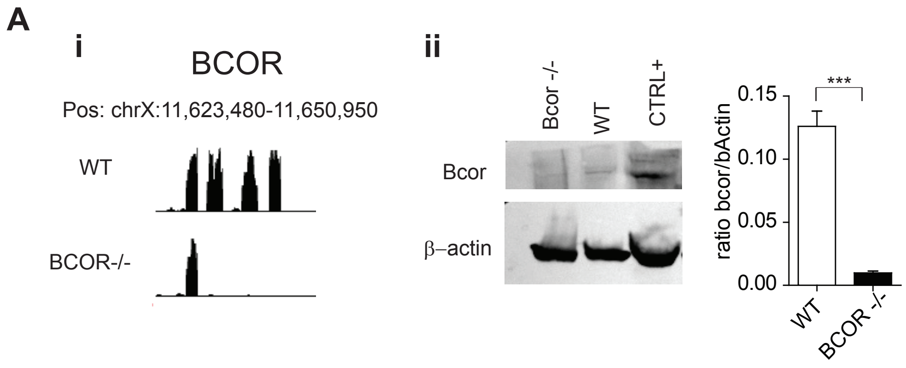

Supplementary Figure 2.

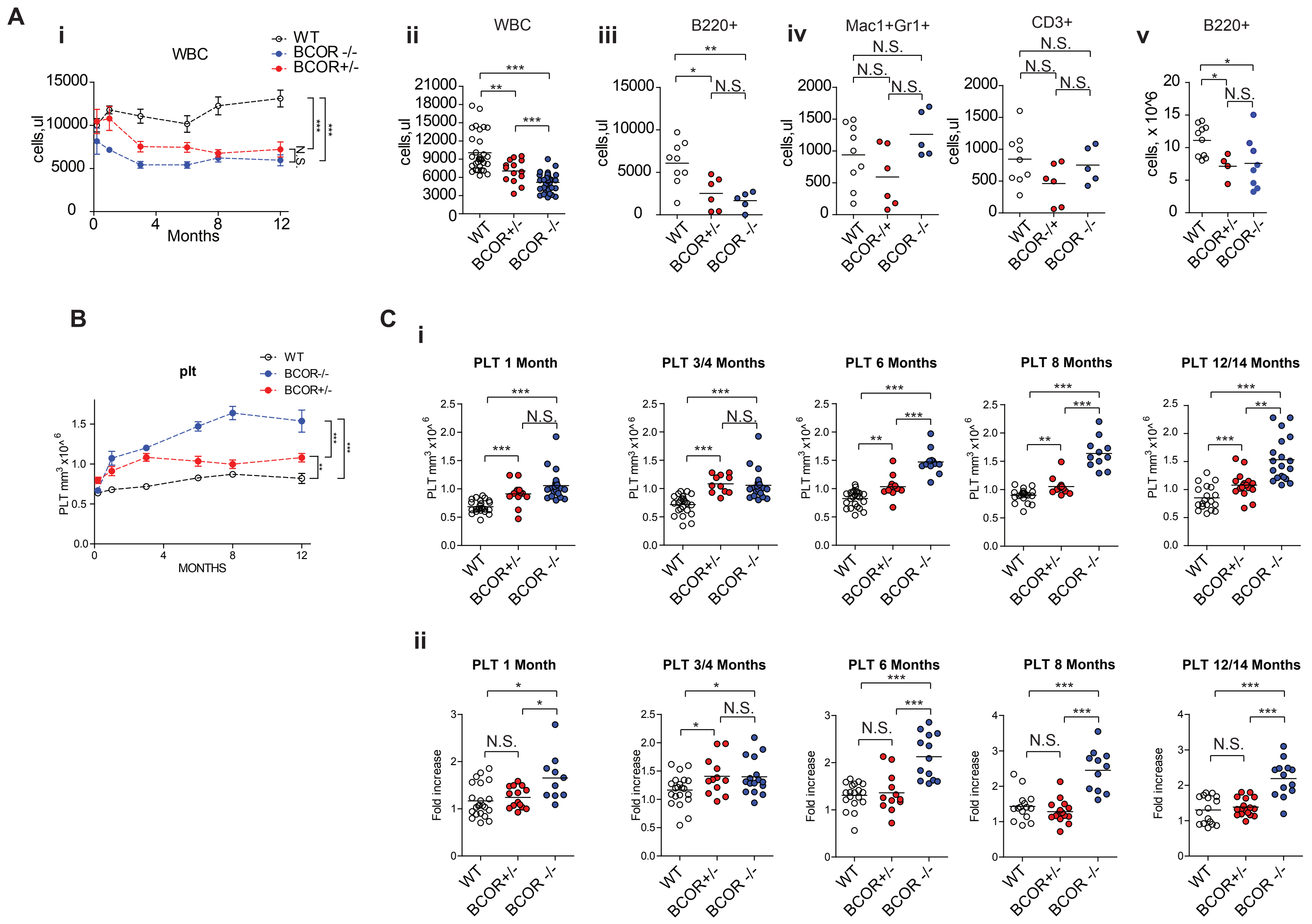

Supplementary Figure 3.

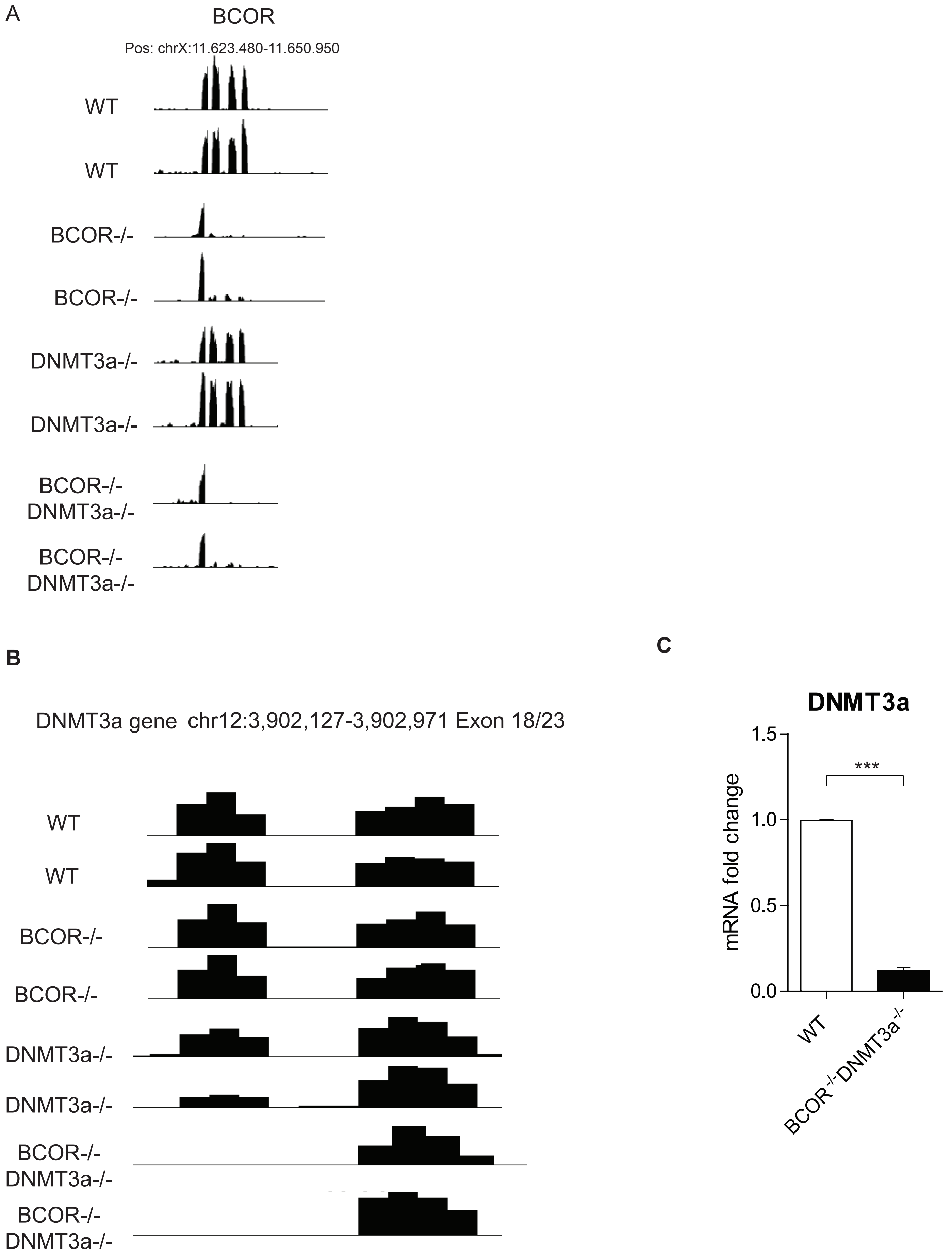

Supplementary Figure 4.

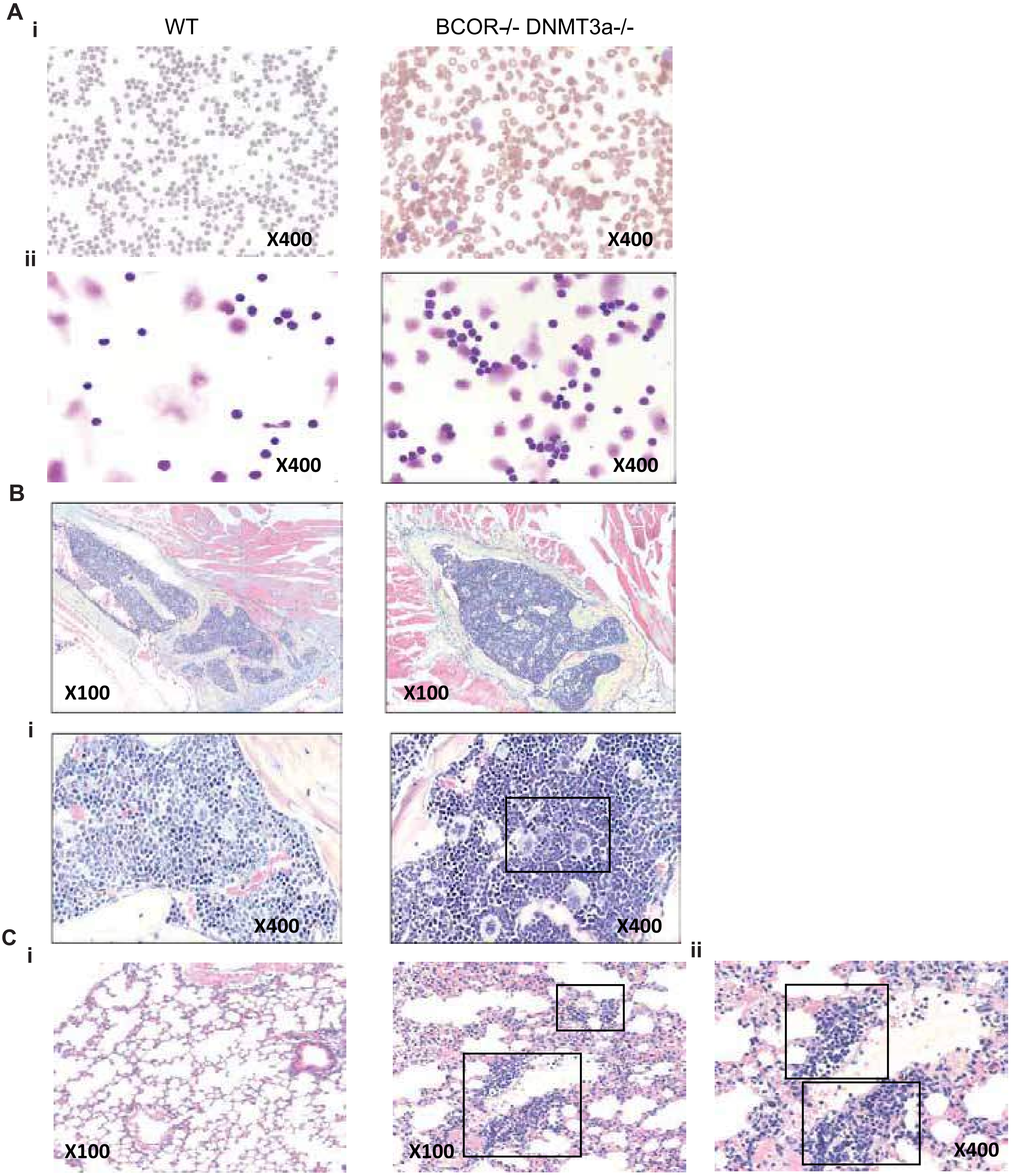

Supplementary Figure 5.

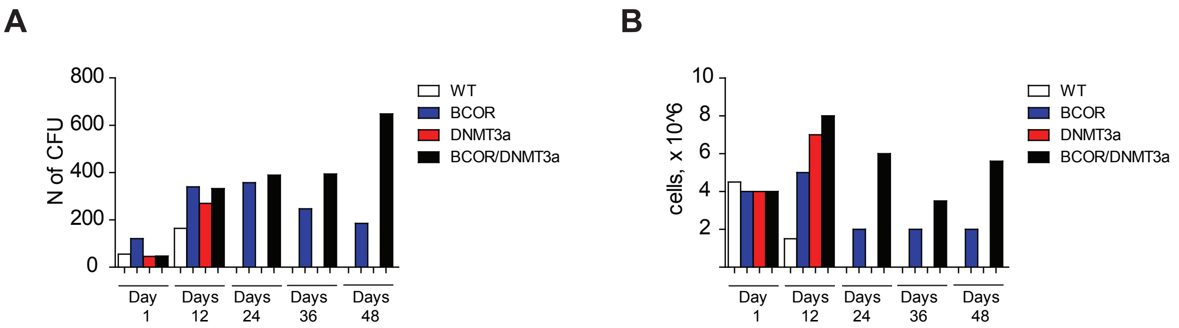

Supplementary Figure 6.

A

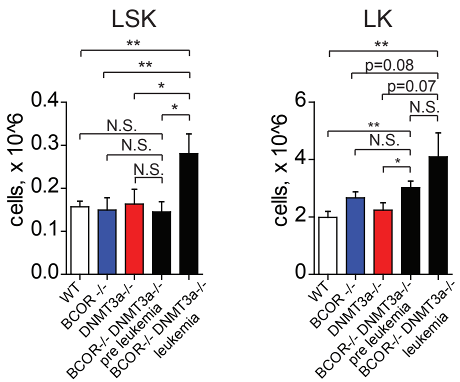

Supplementary figure 7.

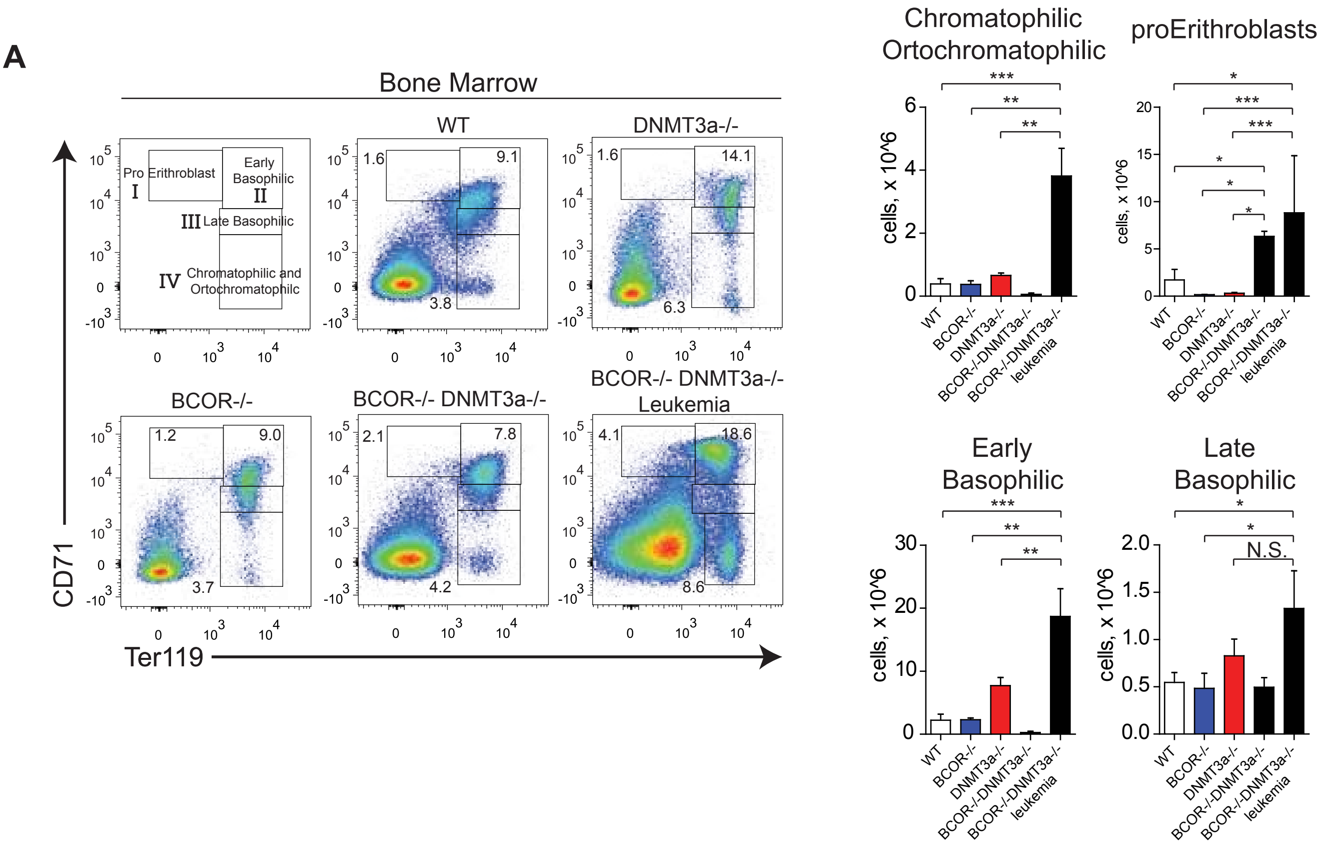

Supplementary figure 8.

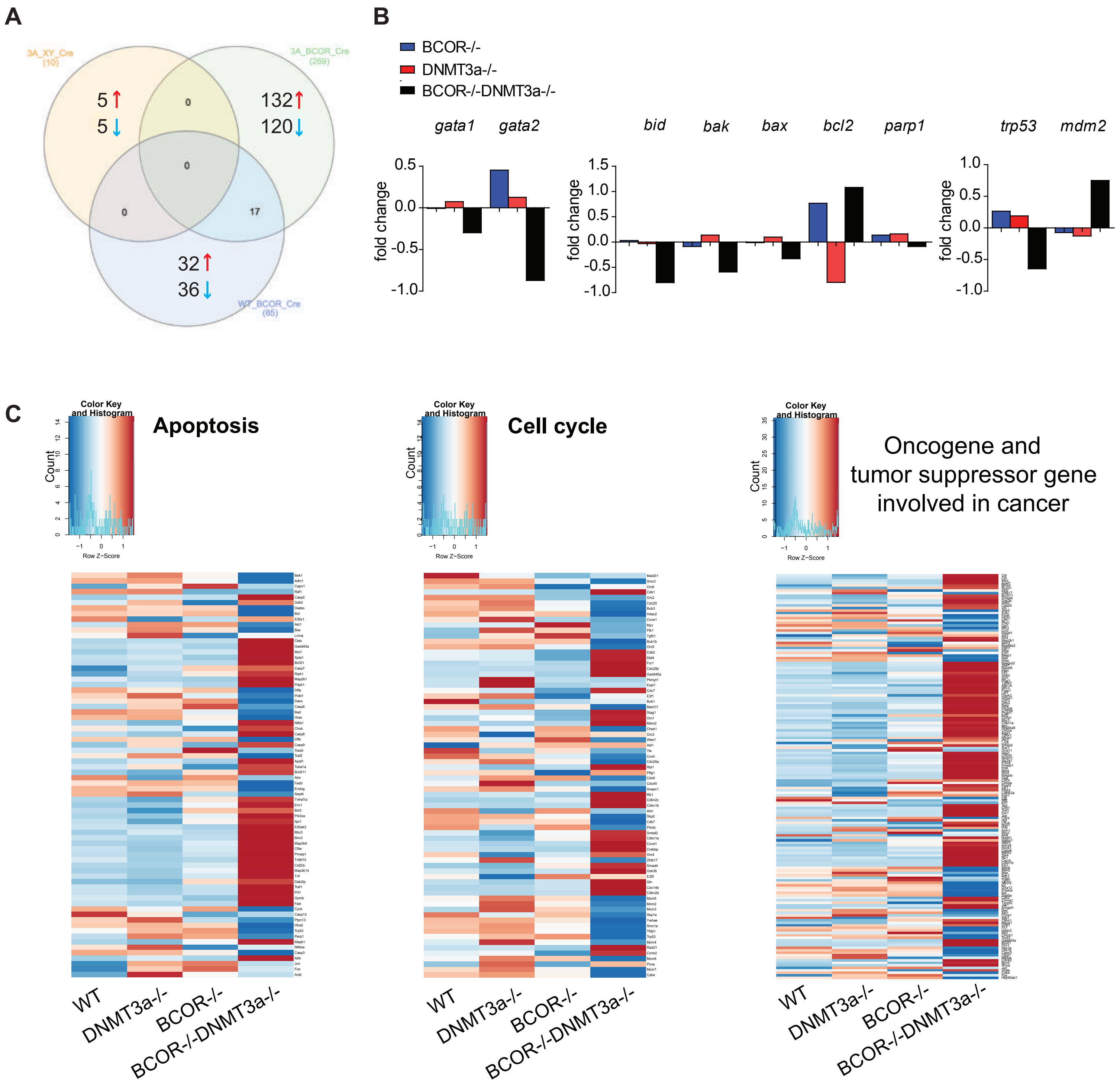

Supplementary Figure 9.

A

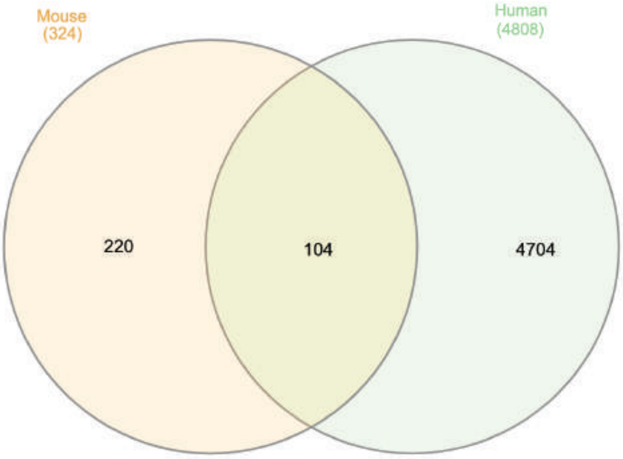

B

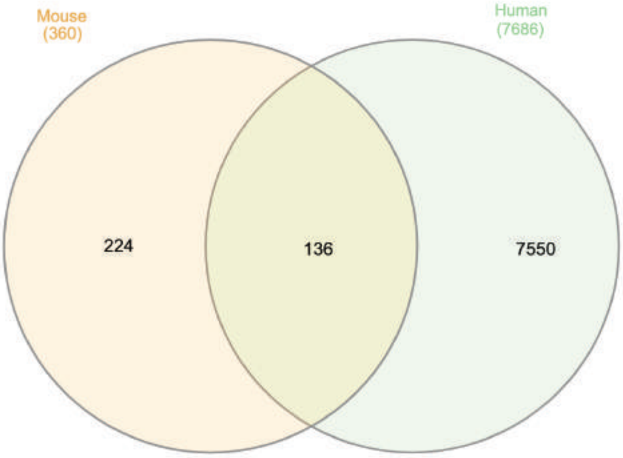

Supplementary Figure 10.

A

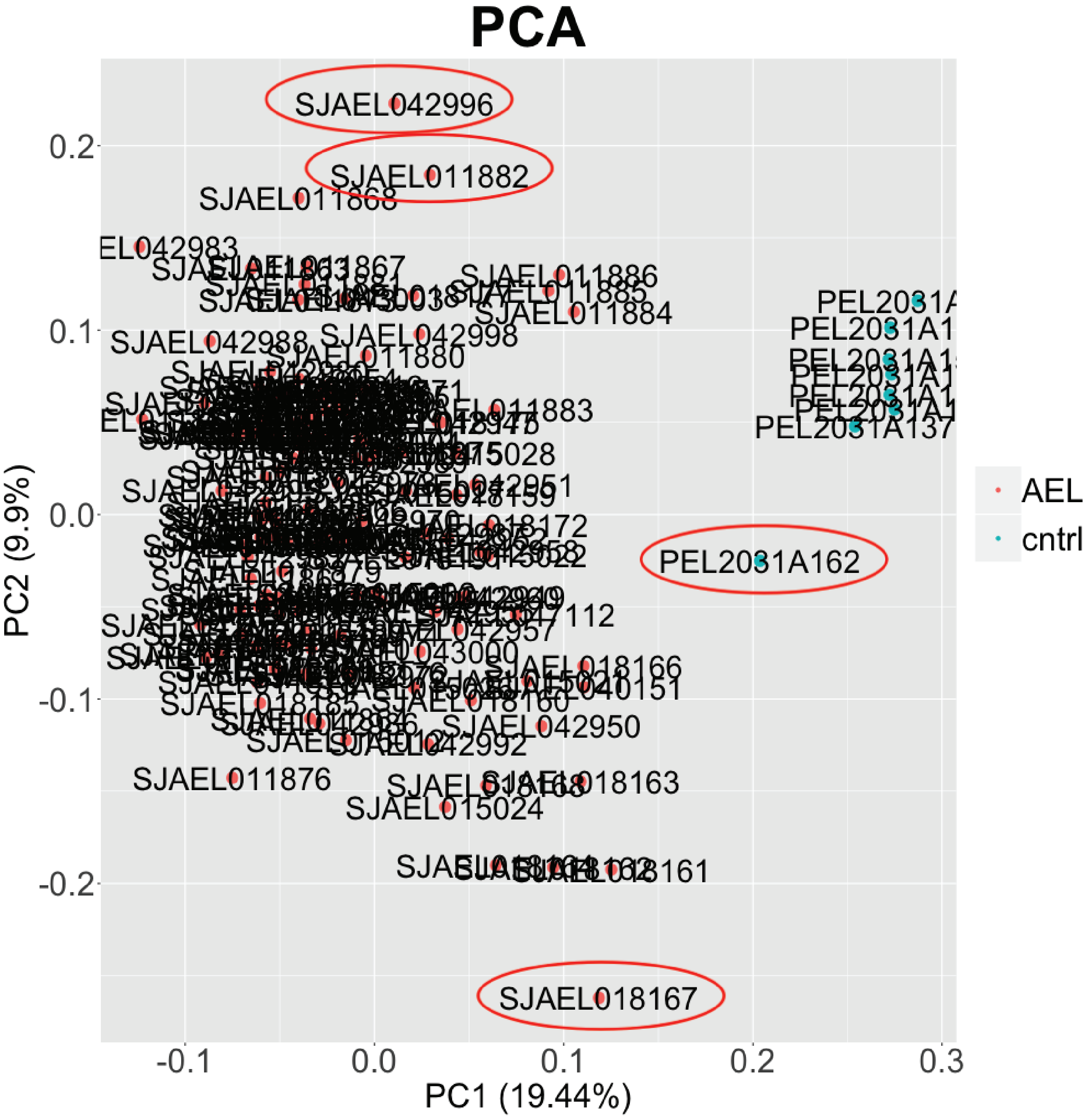

Supplementary Figura 11.

BCOR/DNMT3a CRE+		BCOR CRE+		DNMT3a CRE+	
GENE ID	FOLD CHANGE	GENE ID	FOLD CHANGE	GENE ID	FOLD CHANGE
Cd22	6,051428474	Gm5643	3,891295597	Cd5	1,875942473
Btnl10	5,624047489	Lilr4b	3,416379686	Loxl2	-1,681735849
Hs3st3b1	5,410814883	Aspa	3,115191852	Arg2	-1,769578016
Pou2af1	5,191041588	C030013G03Rik	2,674060508	Klf6	-1,794407679
Rhag	5,099746969	Dusp9	2,66270129	Moap1	-1,972707434
Cldn13	4,681441382	Emp1	2,635018659	Klf2	-2,211169919
Il4i1	4,588355751	Plk2	2,604749555	Spata18	-2,426293898
Skap1	4,57034121	Slco2a1	2,57636708		
Rhd	4,53263566	Thbs1	2,44828131		
Plcd3	4,358039191	Alox15	2,414319691		
Dnase1l3	4,215280285	Slamf1	2,414231523		
Cd38	4,185921322	Gm6307	2,308680079		
Tspo2	3,946306219	Arhgap22	2,305834705		
St6gal1	3,924071205	Cyp11a1	2,300181291		
Sdc4	3,915052328	Dusp5	2,210232778		
Plxdc1	3,888202839	Ccdc30	2,203950222		
Agtr1a	3,707043489	Col1a2	2,198138286		
Cp	3,609202952	Akr1c12	2,16506574		
Ntn4	3,59154995	Car12	2,129680003		
Tulp4	3,409609859	Gem	2,072195549		
Arntl2	3,407305285	Ankrd29	2,060273368		
Mt2	3,33657933	Rgs8	2,058753		
Fn3k	3,19357122	9230110C19Rik	2,046612496		
Epas1	3,066367548	Raver2	2,046104761		
Mpp2	3,055299337	Angptl2	2,041461767		
Ets1	2,994242363	Nlrp1b	2,000692604		
Fam222a	2,946643704	Nlrc3	1,977629847		
Slc22a23	2,945596666	Arhgef9	1,972828005		
Prf1	2,905640791	Uchl1	1,954301853		
Tmem120b	2,820923469	Cdon	1,934162901		
St3gal5	2,796313924	Gprin3	1,931911894		
Cmklr1	2,769208897	Arpp21	1,922938108		
Lefty1	2,75299398	Apoo	1,911385602		
Tmc8	2,675436319	Tert	1,888651417		
Aldh3b1	2,672048132	Ovgp1	1,877939054		
Mgst3	2,627215952	C1qtnf6	1,87504273		
Ppap2b	2,619608325	Dcbld2	1,864916323		
Traf3ip2	2,581165509	Lama5	1,806027992		
Aqp1	2,579383051	Klhl4	1,803649456		
Nxpe4	2,5388189	Tssk4	1,801796766		

Pirb	2,525084868	Gm13154	1,799823583		
Ankrd33b	2,496933148	Fstl1	1,791093983		
Ctss	2,47750782	Itgae	1,790833253		
Ces2g	2,456750437	C1ra	1,783334499		
Spire1	2,448143398	Kcnip4	1,76977694		
Gpc4	2,438148839	Il18r1	1,758422807		
Tmem243	2,404197074	Vash2	1,74145478		
Fam212b	2,396407298	Synpo	1,73876946		
Dennd2c	2,334822391	Pkib	1,732064021		
Ankrd61	2,286262569	Tjp1	1,714130897		
Abcb10	2,245081653	Pcdhb17	1,668774958		
Nrp1	2,235061618	Adamts6	1,654446025		
Asprv1	2,230720629	Vps37d	1,625463151		
2010016I18Rik	2,219212236	Rab36	1,613793788		
Pik3ap1	2,154118255	Rapgef4	1,613240575		
Unc93b1	2,144794187	Gm12657	1,597984146		
Tlr12	2,142345834	Obscn	1,581555174		
Snn	2,049210525	Ifi203	1,573931828		
Gimap7	2,04532921	Tnfrsf10b	1,571793614		
Kif19a	2,034591598	Sgsm1	1,562391288		
Eif2ak3	2,015782984	Gimap5	1,542304396		
Stx11	2,009799893	Cd69	1,534075908		
Gata1	2,000695225	Ar	1,528640512		
Samd14	1,963824095	Btla	1,527080512		
Bcl2l1	1,953521375	Rundc3b	1,52077885		
D8Ertd82e	1,935004237	Lca5	1,514247299		
Gsn	1,9269306	Plxdc2	1,510964692		
H2-T24	1,923396186	Cd33	1,505582193		
Evl	1,902930062	Med21	-1,502485344		
Hemgn	1,899573315	Tlcd1	-1,509657938		
Lnx2	1,896235003	Phyhip	-1,522140431		
Camk2d	1,877510493	Emc9	-1,544302843		
Aff1	1,834640415	Clec1b	-1,573527409		
Vav2	1,832591609	Cebpa	-1,588909669		
Pou2f2	1,807589675	Baiap3	-1,63141075		
Xdh	1,795301395	Mmp14	-1,656587025		
Sgk1	1,793821408	B3gnt8	-1,668211756		
Aldh1a7	1,785119572	Spsb4	-1,681596102		
Pde8a	1,769898593	Wnt10b	-1,68302016		
Irf5	1,769677613	Depdc7	-1,685767691		
B430306N03Rik	1,754641841	Ap3s1	-1,719988113		
Gadd45g	1,75258665	Tubb3	-1,747919289		
Otub2	1,746247559	Slc26a8	-1,75512699		

Dclre1c	1,735503677	Hvcn1	-1,758467741		
H2-Ab1	1,725876559	F630028O10Rik	-1,759555267		
Zbp1	1,6948468	Cxadr	-1,77304009		
Tecpr1	1,692445989	Tlr1	-1,868157945		
Mir6236	1,688595001	Slc4a8	-1,880628665		
Ctsb	1,664651608	Mgst2	-1,904109634		
AA986860	1,632129152	Dab2	-1,950433101		
Traf4	1,628713208	Tnfsf13	-1,963460811		
Cysltr1	1,613836913	Wfdc21	-1,977865936		
B3gat2	1,588186097	Dsp	-2,012923659		
Klf3	1,578631414	Clec4a2	-2,050871594		
Mthfd2	1,559895989	Ltb4r1	-2,055939112		
Dennd4a	1,551899512	Syngr1	-2,06800894		
Neu1	1,550630938	Abcb9	-2,069434008		
Lbh	1,545987602	Cebpd	-2,161119636		
Slc25a38	1,541871481	Ptgir	-2,173358305		
Ubac1	1,537234022	Lin28a	-2,482915978		
Mylip	1,513692033	Trem3	-2,721898823		
Man2a1	1,511907131	G0s2	-3,029256756		
Arhgef12	1,507343819	Fcnb	-3,149815204		
1810011H11Rik	-1,506898758	Ankrd63	-3,992592001		
Stk26	-1,512998696				
Extl2	-1,513477738				
Fgd4	-1,514013314				
Heatr5a	-1,518265889				
Lhfpl2	-1,519159788				
2010204K13Rik	-1,529766345				
Ltbr	-1,532714125				
Gstk1	-1,540624553				
Trip6	-1,544809865				
4921507P07Rik	-1,552222925				
Dmwd	-1,553137236				
Vkorc1	-1,558658149				
Ifitm2	-1,558766094				
Zfp763	-1,560288841				
Xpc	-1,565111297				
P3h1	-1,569818149				
Galm	-1,570012405				
Guca1a	-1,573371669				
Tmem231	-1,584783954				
Vsig10l	-1,587671007				
Padi4	-1,591238719				
Cryz	-1,593490518				

Ccdc40	-1,59432913				
Treml2	-1,597706454				
Enpp5	-1,598189567				
Aldh7a1	-1,599516118				
Jpx	-1,601029337				
Rbpms	-1,602012407				
Tead2	-1,606809602				
Rasl12	-1,607465264				
2900005J15Rik	-1,609109843				
Gatsl2	-1,614901304				
1500009L16Rik	-1,616723267				
Rorc	-1,617174219				
Irak1bp1	-1,617594455				
Syde1	-1,617791783				
Tmem205	-1,621526498				
Gabarapl1	-1,622860693				
Zc4h2	-1,626833461				
Pbx4	-1,627947031				
Rab11fip3	-1,628677051				
Setmar	-1,63422751				
Pcyox1l	-1,637585625				
Hsd3b7	-1,643266706				
Ugt1a7c	-1,648703912				
9930014A18Rik	-1,648875308				
Atp6v1g2	-1,652858083				
Tle2	-1,653312325				
Atg4c	-1,654387005				
Dfna5	-1,658216794				
4930520O04Rik	-1,658994773				
Rec8	-1,659224533				
Large	-1,661332442				
Macrod1	-1,664198832				
Dhdh	-1,675686222				
Paqr7	-1,679872204				
4931428F04Rik	-1,687039502				
Klhl3	-1,68743743				
Tmem181b-ps	-1,688493599				
Megf8	-1,692441744				
Tet1	-1,697583293				
Flt3	-1,701060035				
Sirt3	-1,703305016				
Gpc2	-1,706184814				
Gm5595	-1,708016196				

Igdcc4	-1,713658425				
Cpq	-1,717492174				
I730030J21Rik	-1,733300359				
Trim47	-1,746015634				
Amot	-1,746540056				
Acsl3	-1,748881638				
Vegfc	-1,750122777				
Fscn1	-1,759777677				
Cacna1a	-1,760428214				
Nos1ap	-1,770513193				
Msl3l2	-1,774553112				
Frmd7	-1,780397388				
Lrrc27	-1,799040472				
Supt3	-1,799908001				
D5Ertd605e	-1,806622315				
Pank1	-1,807270862				
Adgrd1	-1,830687533				
Ngfrap1	-1,835256059				
Pcdhgb4	-1,836259858				
Mthfd2l	-1,847115757				
Acot7	-1,849991031				
Pak1	-1,85799089				
Tfr2	-1,860762028				
Ybx2	-1,862847087				
Tcp11	-1,863073086				
Mtss1l	-1,865669191				
Kifap3	-1,878409617				
Itm2a	-1,881105081				
Kcnd1	-1,882875402				
Ltc4s	-1,885470699				
Lmo2	-1,889861965				
B230118H07Rik	-1,90164268				
Efr3b	-1,902695763				
Neto2	-1,910605137				
Deptor	-1,911583726				
Ccl27a	-1,913398126				
Adrbk2	-1,927439938				
Gm7694	-1,928492831				
Ocrl	-1,932424757				
Pcp4l1	-1,94364981				
Kctd1	-1,956493121				
Rai14	-1,958757616				
Pemt	-1,965476717				

Tcf15	-1,973992638				
Snx7	-1,987553442				
Scamp1	-1,988209098				
Itih5	-2,005844003				
Gm15910	-2,008615844				
Med12l	-2,018230574				
Ispd	-2,02782076				
Syngap1	-2,036377806				
Scube3	-2,044562725				
Lrfn4	-2,044809506				
Smo	-2,049219757				
Eid2	-2,055757529				
Dlg4	-2,057721937				
Glis2	-2,062932216				
Gjb3	-2,066060274				
Tspan4	-2,067561929				
Rnf180	-2,069424608				
Ggt5	-2,071605629				
Fsd1l	-2,075645871				
Mfge8	-2,07970996				
Lonrf3	-2,092714541				
Mylk	-2,100232434				
Slc25a43	-2,104264801				
Tceal8	-2,110035658				
Zfp286	-2,111085375				
Csf3r	-2,122871431				
Nlgn2	-2,129168503				
Hook1	-2,13052496				
Rabl2	-2,13867182				
Mb21d2	-2,138772107				
Ccdc149	-2,139016189				
Mansc1	-2,140945018				
Decr1	-2,141137786				
Armcx3	-2,142223194				
Lekr1	-2,147671609				
Kcnab1	-2,150037465				
Def8	-2,153270329				
5730409E04Rik	-2,176743289				
Spred3	-2,193968142				
Slc16a7	-2,194966807				
Slc24a3	-2,211381352				
Tacstd2	-2,213818395				
Upp1	-2,216255926				

Dctd	-2,217250816				
Them7	-2,218325611				
Myl10	-2,219458009				
Nav1	-2,223188558				
Kdelc2	-2,223678516				
Zfp937	-2,226183234				
Smim10l2a	-2,23679037				
Hacd4	-2,240133951				
Dnmt3b	-2,242024992				
Pglyrp2	-2,248197902				
Mtfr2	-2,249578834				
Efna4	-2,253218926				
Samsn1	-2,254635741				
Camk1	-2,260058054				
Aldh5a1	-2,260912392				
Tcp11l1	-2,264311844				
Anxa4	-2,264552197				
Dapk1	-2,26841476				
Kit	-2,269918647				
H1fx	-2,279516407				
Dapp1	-2,281578163				
Npl	-2,32312586				
Slc22a17	-2,323849296				
Fam171a2	-2,324353729				
Clec4d	-2,325594542				
Ctxn1	-2,328269074				
Gnat2	-2,33857606				
Psma8	-2,345248799				
Zfp608	-2,367899141				
Bbs7	-2,36814161				
Phldb2	-2,368363152				
Ica1	-2,370460417				
Npr2	-2,376918733				
Fam131a	-2,384070239				
Vdr	-2,390316489				
Rbpms2	-2,409356961				
Dock7	-2,41054075				
Gnb5	-2,4130371				
Ak4	-2,419557649				
Osbpl1a	-2,420568265				
Tmem40	-2,42074159				
Zfp184	-2,42329581				
Bicd1	-2,443549322				

Fam110b	-2,449157055				
Ikzf2	-2,449469345				
4930515G01Rik	-2,451370353				
Tdrkh	-2,461410965				
Rgs18	-2,46625499				
Arhgap32	-2,467656558				
Fabp5	-2,474098766				
Slc25a27	-2,475240959				
Egln3	-2,476099313				
Sema4g	-2,47686237				
Sdsl	-2,480182433				
Zfp30	-2,48102548				
Zfp248	-2,492582832				
Clic5	-2,498141365				
Prdm16	-2,499100869				
Tfec	-2,499323299				
Snx31	-2,502030709				
Maged2	-2,502940961				
Clnk	-2,506332419				
Adgra2	-2,511854177				
Tas1r1	-2,517631913				
Kcnk12	-2,519975013				
Ociad2	-2,521085774				
Sall2	-2,530193645				
Ndrg1	-2,534492084				
Ms4a2	-2,541193888				
Nudt12	-2,544655584				
Gprc5b	-2,549743415				
Rbp1	-2,552536148				
Irf6	-2,553364609				
Ptrf	-2,553563335				
Ier3	-2,557075164				
Pcdhgb6	-2,564191403				
Rusc2	-2,57077207				
Adam22	-2,578712134				
Fsbp	-2,586128129				
Tpmt	-2,593686421				
Fam92a	-2,599436596				
Lincred1	-2,60020807				
Hspa12b	-2,609081408				
Ccl9	-2,620505352				
Shank3	-2,629899383				
Six5	-2,632527391				

Il17rc	-2,634770857				
Slco3a1	-2,650318345				
Ccm2l	-2,659052934				
Copz2	-2,666375721				
Apln	-2,667217002				
Fam169a	-2,6711201				
Zfp532	-2,673089314				
Camk2a	-2,673464929				
Plag1	-2,682220774				
Tmem14a	-2,682430565				
P3h3	-2,68354975				
Il1r1	-2,686583341				
Mmp15	-2,686958558				
Ccdc112	-2,688990145				
Pde4c	-2,719742706				
Gm14420	-2,72244453				
Slc7a4	-2,726437105				
Dnaaf3	-2,755598853				
Zfp521	-2,757813621				
Bex6	-2,766385107				
Selp	-2,766558956				
Kcnh2	-2,773983036				
Rtn4r	-2,775254564				
Cd27	-2,776589716				
Ltbp3	-2,776625045				
Pkd2	-2,777283658				
Pter	-2,780369889				
Slc24a5	-2,788787983				
Cdk18	-2,795809518				
Fgd1	-2,796550219				
Pafah2	-2,797468127				
Fgfr3	-2,803628626				
Zfp109	-2,823123627				
Mycbpap	-2,824038892				
Hoxa9	-2,839698907				
Epha7	-2,848029583				
Abca4	-2,849042665				
Vash1	-2,855583352				
Gspt2	-2,875726357				
Cxx1c	-2,880213935				
Zc2hc1a	-2,882785164				
Slc18a1	-2,891582815				
Hoxa10	-2,905711362				

Cyp2j9	-2,918871356				
Zfp57	-2,934417448				
Rab17	-2,943610375				
Rbm11	-2,960597936				
Prr36	-2,964225859				
Gm973	-2,976745522				
Akr1e1	-2,999505319				
Tmem215	-3,005859705				
Afap1l1	-3,0118149				
Nkx2-3	-3,021908797				
Maoa	-3,024798667				
Sorbs3	-3,032738104				
Clstn3	-3,035451876				
Ephb6	-3,038052341				
Tmem121	-3,040723387				
Pdgfrb	-3,05759197				
Hgf	-3,059208157				
Ccdc158	-3,062474782				
Arhgap6	-3,068011413				
Slc25a13	-3,088571252				
Fkbp9	-3,092250605				
Tcam1	-3,117906959				
Ehbp1	-3,123810174				
Gata2	-3,138656162				
Slc18a2	-3,140052334				
Slc27a6	-3,14592089				
Ttc8	-3,149256459				
Il15	-3,160482267				
Ecscr	-3,171821098				
Ifitm1	-3,179178662				
Il31ra	-3,182984109				
Stxbp4	-3,197134687				
Sema3d	-3,201049686				
Serf1	-3,203394304				
Hgfac	-3,208798819				
Nlrp10	-3,211994109				
Procr	-3,22265641				
Tmem98	-3,227448566				
Obsl1	-3,231519864				
Adgrg1	-3,233298106				
Ppp1r26	-3,236722697				
Tmem159	-3,237574628				
Cldn12	-3,252521135				

Esam	-3,252927131				
Rbfox2	-3,261326668				
Muc13	-3,266924277				
5930403L14Rik	-3,267099074				
Rab38	-3,272881208				
8430408G22Rik	-3,281653613				
Kcng1	-3,285900275				
Pbx1	-3,28706641				
Camsap3	-3,2881548				
Ppp2r3a	-3,309748795				
Zfp93	-3,315437267				
Cyp7b1	-3,33426628				
Col16a1	-3,337169606				
Nhsl1	-3,340909043				
Dach1	-3,343522				
Hnmt	-3,346141506				
Ppp1r9a	-3,35186361				
Mamdc2	-3,364483627				
Trpc6	-3,402102751				
Fam171a1	-3,409760024				
Fut7	-3,432218826				
Lypd6b	-3,441383775				
Gcnt2	-3,446547249				
Gng11	-3,458410142				
Paqr8	-3,459831718				
Fzd3	-3,500068317				
Pld6	-3,515432808				
Mycn	-3,516599612				
Tmprss7	-3,522672274				
Adgrg7	-3,52572204				
Alpk3	-3,529398277				
Tmem150b	-3,579980493				
Scml2	-3,589866832				
Cdcp1	-3,599880036				
Fzd8	-3,603354031				
Spns2	-3,619394834				
Brsk1	-3,623800272				
Aif1l	-3,624864769				
Efcc1	-3,652405817				
Slc16a11	-3,665930324				
2610305D13Rik	-3,673037951				
Arhgef28	-3,674781819				
Ube2e2	-3,687709878				

Prex2	-3,693095748				
Vldlr	-3,708183945				
Cpa3	-3,729930988				
Lhcgr	-3,731437572				
Rhobtb3	-3,743011494				
Cc2d2a	-3,749775606				
Ttpa	-3,765600134				
Dcaf12l1	-3,772844577				
C030034L19Rik	-3,788007434				
H2afy2	-3,789377868				
Ccdc8	-3,796488367				
Slc22a18	-3,804926888				
Meis1	-3,809527481				
Nap1l3	-3,81538611				
Gcsam	-3,830594686				
Cadps2	-3,863020388				
Cyp2j6	-3,864710312				
Mdga1	-3,876694549				
Mdfi	-3,899181037				
F2rl3	-3,908211334				
Gucy1a3	-3,91012318				
Mpl	-3,919104647				
Nrxn1	-3,92627918				
Nwd1	-3,943705186				
Armcx4	-3,963057155				
Slc16a12	-3,997025183				
Nynrin	-3,99826319				
Casp12	-4,016426533				
Gprasp2	-4,04703371				
9030619P08Rik	-4,224524535				
Napb	-4,224885435				
Hmga2	-4,249797183				
Otos	-4,289877733				
Tek	-4,335160982				
Il17re	-4,34582444				
Hnf4a	-4,355656703				
Ddx4	-4,376884331				
Prkg1	-4,426263459				
Armcx1	-4,462947709				
Hlf	-4,466472172				
Mecom	-4,512791493				
Btc	-4,525944228				
Edar	-4,571956294				

Tie1	-4,574175316				
Myct1	-4,619845714				
Angpt1	-4,642006156				
Mc5r	-4,709210782				
Cd34	-4,756717133				
Adgrl4	-4,978389548				
Cacnb2	-5,057105934				
Slc22a3	-5,146233063				
Layn	-5,215136332				
Tnip3	-5,523606351				
Pkia	-5,646131855				
Zfp2	-5,923495215				
2900008C10Rik	2,588859903	2900008C10Rik	-1,988454007		
Hck	2,199588944	Hck	-1,583734761		
Gimap8	1,684412837	Gimap8	1,778695551		
Ric3	-1,707138871	Ric3	1,59922536		
Ccl4	-1,945043308	Ccl4	1,708548564		
Fzd9	-2,085927857	Fzd9	1,614323974		
Klhl30	-2,432873095	Klhl30	1,776449388		
Ccl3	-2,487867526	Ccl3	1,541416384		
Cpxm1	-2,587271296	Cpxm1	1,588277842		
Atp8b5	-2,632841699	Atp8b5	1,925549561		
Myo5c	-2,650052795	Myo5c	2,302114181		
Rgs1	-2,684595157	Rgs1	1,792435215		
Capsl	-2,694759268	Capsl	2,226428335		
Plscr4	-2,702183667	Plscr4	1,854640171		
Gstm7	-2,754141125	Gstm7	1,912277501		
Dntt	-2,880139795	Dntt	1,574630278		
Clec4e	-2,90418373	Clec4e	2,002399		
Fam135a	-2,907403046	Fam135a	1,748158887		
Ctla2a	-2,93469143	Ctla2a	1,52498167		
4930452B06Rik	-3,121800618	4930452B06Rik	4,094473243		
Apcdd1	-3,154751729	Apcdd1	2,290770075		
Morn4	-3,20768279	Morn4	1,705897245		
Ttc12	-3,222317239	Ttc12	2,310801044		
Tfcp2l1	-3,356825084	Tfcp2l1	1,805795372		
Robo4	-3,598791354	Robo4	1,613216949		

Table S1: List of differentially expressed genes in the LSK BM cells of BCOR/DNMT3a, BCOR and DNMT3a mice compared to WT controls.

BCOR/DNMT3a		BCOR		DNMT3a	
ID GENE	LOG FOLD CHANGE	ID GENE	LOG FOLD CHANGE	ID GENE	LOG2 FOLD CHANGE
Gm21975	6,832263445	Il4i1	5,442056048	Prg2	2,216110076
6430548M08Rik	5,024646945	5930403L14Rik	3,80258118	Vcam1	1,868791436
S100a6	4,814028267	2900008C10Rik	3,237230566	Pcdhga5	1,7434035
Slfn2	4,393534463	Nkx1-2	2,949192458	Rnls	1,711680536
Slfn4	4,314318743	Itga9	2,705001108	Nacad	1,693088783
Clec4a2	4,245449978	Hoxa10	2,55211065	Catsper2	-1,68796076
Ccr7	4,105442913	Prdm16	2,448756132	Iigp1	-1,799279557
B430306N03Rik	4,066108058	B4galt6	2,343327255	Gm15446	-2,057893846
Zc3h12d	3,92602666	Cebpa	2,259167507	Gdpd3	-2,175651432
Filip1l	3,794707703	Fgf3	2,132347415	Klra4	-2,638044932
Tlr9	3,75080541	Peli2	2,107803225		
Gbp8	3,719035215	Cldn15	2,083019534		
App	3,640319432	Hoxa7	1,94972639		
Fcrla	3,627953945	Rab6b	1,925824627		
Rhoc	3,548746364	Hmga2	1,783164286		
Pirb	3,436651945	B3gnt7	1,747769872		
Rin2	3,401133771	Pcdhgc3	1,741183684		
Map3k8	3,387716471	Mira	1,736849316		
Ptpn22	3,269164784	Ak4	1,729611733		
Pqlc3	3,232934918	Ctbp2	1,722066037		
Hck	3,192709471	Klf2	1,700057858		
Fam102a	3,157622644	Src	1,699773001		
Il1r2	3,148843319	Ms4a3	1,694426308		
Tnfrsf13b	3,127629936	Slc9a2	1,680035571		
Jdp2	3,059083659	A430105I19Rik	1,665316921		
Lrrc16a	3,019865869	Zfp516	1,658088203		
Fcer1g	2,916698526	Cap2	1,645596738		
D330041H03Rik	2,891592291	Slc35e4	1,638924977		
A630033H20Rik	2,876925526	Slc35f2	1,617437985		
Nucb2	2,872917624	Hoxa9	1,613901608		
Gpr65	2,863304403	Gkap1	1,567970627		
Il12rb1	2,853924827	Wdr78	1,520169522		
Csf2rb	2,845033855	Ndrg2	-1,502648756		
Sh2d1b1	2,834767252	Clock	-1,536576273		
Cd247	2,833045969	1700048O20Rik	-1,628482819		
Degs2	2,799176218	Akap2	-1,64511209		
Dram1	2,735645992	Xlr4c	-1,681385693		
Lck	2,691037006	Hif3a	-1,684656569		
Irf2bpl	2,68788063	Slc36a4	-1,720513588		
Ly75	2,68462492	Axl	-1,730626532		
Appl2	2,670636474	Vwa7	-1,736990502		

Lag3	2,650188379	Clgn	-1,74532398		
Pld4	2,639579467	Bdh1	-1,775144198		
Hdc	2,631745643	Gm15787	-1,856720252		
Mylip	2,622542221	Fabp5	-1,981971402		
Stk39	2,605675049	Scin	-1,985453824		
Ctsh	2,575755639	Mmp8	-1,993329613		
Il4ra	2,570099789	Tspan17	-2,006867067		
Padi2	2,567649694	Plk2	-2,019739267		
Bhlha15	2,489489906	Prnp	-2,033677513		
Gpr137b-ps	2,439378607	Mylk3	-2,045068559		
H2-Q9	2,416797827	Slc30a10	-2,088683098		
Epsti1	2,367855567	Raver2	-2,126894291		
Tmem140	2,328671974	Tjp1	-2,166383698		
Rogdi	2,312122769	Sdc2	-2,180520272		
Nab2	2,237696458	D330045A20Rik	-2,323084912		
Adora2a	2,231224401	Dkkl1	-2,386688833		
Mefv	2,228755988	Colq	-2,446142227		
Rab39	2,227101683	Chil1	-2,516665054		
Gem	2,209775542	Ly6d	-2,556753199		
Gpr174	2,209316067	Dapk2	-2,623432162		
Trps1	2,198351147	Susd2	-2,640618364		
Tbkbp1	2,1874883	Slc52a3	-2,765941193		
Tcf7	2,186460138	Mmp9	-2,774278143		
Hivep2	2,151339065	Retnlg	-2,849167854		
Cd180	2,120873328	Gpr141	-3,064263635		
Birc3	2,085032704	Spry4	-3,811634086		
Zbtb4	2,084199829	Klra18	-7,60293883		
Tnf	2,07236756				
Gabbr1	2,045022764				
Il6ra	2,038921186				
Gadd45b	2,035618014				
Cpd	2,02616411				
Fkbp1b	2,007333302				
Ms4a6c	2,004350709				
3830408C21Rik	1,987600728				
Rbpms	1,957277047				
Itgal	1,949220342				
Bspry	1,940342502				
Bcl6	1,937554353				
D430020J02Rik	1,935144592				
Cdk14	1,912944682				
Dennd1c	1,909989599				
Aph1b	1,907284027				

Ptprj	1,890514882				
Plekha1	1,888195157				
Mturn	1,887579649				
Ovgp1	1,885087922				
Akr1c12	1,877420537				
Cytip	1,876410487				
Prr5	1,861356476				
Pkp3	1,855211125				
Bcl9l	1,854894953				
Micall1	1,841330561				
H2-K2	1,827299495				
S1pr1	1,824845978				
Relb	1,801693717				
Shisa5	1,799236653				
Zswim6	1,792311331				
Dcbld2	1,790498512				
Cd2ap	1,777664252				
Tnks1bp1	1,776545467				
Gramd3	1,767454213				
Cpne3	1,766725359				
Nlrc4	1,759896958				
Alcam	1,720413788				
Rasgrp4	1,716404664				
Fam105a	1,714021967				
Cd274	1,704852788				
Fndc9	1,665277832				
Trove2	1,661874466				
Atp8b4	1,654697095				
Mllt6	1,64003424				
Alox5ap	1,639044444				
Notch1	1,630659692				
Tnfrsf1b	1,609400479				
Mef2a	1,601483716				
Diap2	1,590562671				
Myadm	1,584790333				
Gm13051	1,576498621				
1700049G17Rik	1,568376879				
Zbtb37	1,566947428				
Pmaip1	1,561555607				
Lmbr1l	1,559560527				
Herc3	1,538603162				
Lats2	1,533314682				
S100a11	1,530040665				

Myo1g	1,528187445				
N4bp3	1,51794328				
Tbc1d2	1,516383587				
B630005N14Rik	1,512786683				
Kctd11	1,505546238				
Adamts3	-1,50002051				
Pter	-1,502859537				
Tuft1	-1,517149199				
Grhpr	-1,523474756				
Dctd	-1,525156652				
Lhfpl2	-1,527083008				
5730460C07Rik	-1,532877966				
Pfn4	-1,546733185				
Frat2	-1,557176212				
Slc25a13	-1,560675257				
Rab26os	-1,57109001				
Mgmt	-1,571482981				
Gorasp1	-1,572169482				
Anxa9	-1,572195049				
Dnph1	-1,583467149				
Mthfd2l	-1,597822723				
Pm20d2	-1,598561281				
Macrod1	-1,619879188				
Rab4a	-1,630804457				
Cnr2	-1,635224586				
Gls2	-1,644507141				
Wdr60	-1,647941593				
Pcyox1l	-1,648692363				
Nthl1	-1,653016277				
4933431E20Rik	-1,679805428				
Selenbp1	-1,682795629				
Acyp2	-1,705604053				
Hook1	-1,72090668				
Rangrf	-1,726518121				
Sdsl	-1,728946756				
Pdk2	-1,751540545				
Gnb4	-1,755141993				
Zfp518b	-1,764967333				
Il1rl2	-1,777981835				
Trip6	-1,790950171				
Ptpdc1	-1,812912076				
Rac3	-1,814330833				
Psma8	-1,83089864				

Lrig1	-1,84585262				
Adgrl1	-1,847493944				
Nfia	-1,889112184				
Angptl4	-1,90489061				
Trib2	-1,912523917				
Acsl6	-1,915940384				
Gm11837	-1,940744059				
Ispd	-1,952811428				
Bmp1	-1,962379577				
Decr1	-1,967025558				
Rasgef1c	-1,973869179				
Aqp11	-1,983152622				
Cpt1c	-1,988108908				
Dapk1	-1,988899173				
Akr1e1	-1,992589319				
Csrp3	-1,995506107				
Igsf3	-2,003211991				
Gm14420	-2,028163808				
Atp7b	-2,060042329				
Tfr2	-2,062166989				
Prelid2	-2,106164092				
Arhgef25	-2,108533688				
Rusc2	-2,11583695				
Hpn	-2,134566854				
Plekha6	-2,135847995				
Cxx1c	-2,156801806				
Nudt12	-2,160814894				
Garem	-2,174252817				
Ociad2	-2,184804227				
Eda	-2,198025396				
Macrod2	-2,201807478				
Aldh1a7	-2,245528221				
Il1rl1	-2,264627636				
Slc7a4	-2,26856121				
Epha7	-2,273519616				
Arhgap6	-2,280168533				
Pdia5	-2,293609745				
9130008F23Rik	-2,320538869				
H1fx	-2,358063751				
Ehbp1	-2,368846162				
Spire2	-2,390951456				
Gnaz	-2,412902953				
Angel1	-2,437788076				

Rnf17	-2,465991594				
Lcat	-2,480401339				
Scml2	-2,539196796				
Top1mt	-2,563036713				
Grb10	-2,5689685				
Mpped2	-2,57447003				
Slc22a3	-2,589686939				
Flywch2	-2,616494299				
Armcx2	-2,628533819				
Chst10	-2,691579821				
Lmcd1	-2,700323504				
Armcx3	-2,703689177				
Armcx1	-2,704627871				
Fam132a	-2,725003666				
BC051537	-2,735838602				
Slc26a1	-2,81265105				
Prss50	-2,814385152				
Pvrl2	-2,867546123				
Tdrkh	-2,905125844				
Zfp2	-2,909921652				
Vstm5	-2,954346174				
Plvap	-2,971742227				
Tmem17	-2,997237029				
Clstn3	-3,029252975				
Sh2d4a	-3,074435404				
Slc18b1	-3,096361843				
Mtap7d3	-3,113754465				
Fkbp9	-3,123377335				
Nags	-3,355777373				
Dntt	-3,433819005				
Il31ra	-3,54752278				
Armcx6	-3,616690113				
Ptpn13	-3,668817063				
Ccdc68	-3,681614115				
Zfp583	-3,696825343				
Sytl4	-4,121464795				
Zg16	-4,233992958				
Rgs9bp	-4,305988861				
Khdrbs3	-5,629204643				
Hopx	2,345271103	Hopx	-2,344072738		
Lgals7	-1,636086762	Lgals7	-2,040450891		
Asns	-1,784195226	Asns	-1,851857433		
Slc2a8	-1,832210868	Slc2a8	-1,594876159		

Maoa	-1,914954048	Maoa	-2,384272602		
Meiob	-2,020971961	Meiob	-1,569583432		
Hmgcs2	-2,314922279	Hmgcs2	-2,789336204		
Plekha8	-2,45548982	Plekha8	-1,704617561		
Gsta4	-2,484415947	Gsta4	-1,718822399		
Zdhhc2	-2,677768348	Zdhhc2	-3,493806875		
Ltbp1	-3,145541092	Ltbp1	-2,272428821		
Ephx2	-3,409325421	Ephx2	-3,173159244		
Synpo2	-3,551785069	Synpo2	-2,0072338		
Bbs7	-3,680718263	Bbs7	-1,870235278		
Atp4a	-4,15929363	Atp4a	-3,283997789		
Ranbp17	-4,279099461	Ranbp17	-2,99137887		
Spon2	-4,874191693	Spon2	-1,830830757		

Table S2: List of differentially expressed genes in the MEP BM cells of BCOR/DNMT3a, BCOR and DNMT3a mice compared to WT controls.
